# From root to result: Portable NIRS-based non-destructive prediction of cassava quality traits

**DOI:** 10.1371/journal.pone.0337761

**Published:** 2025-12-03

**Authors:** Paulo Henrique Ramos Guimarães, Cinara Fernanda Garcia Morales, Tamires Sousa Cerqueira, Marcos de Souza Campos, Eder Jorge de Oliveira

**Affiliations:** Embrapa Mandioca e Fruticultura, Cruz das Almas, Brazil; University of the West Indies at Cave Hill, BARBADOS

## Abstract

Cassava (*Manihot esculenta* Crantz) is a staple food and a key industrial crop across tropical regions, but traditional phenotyping for critical quality traits like dry matter content (DMC) and starch content (StC) is a laborious and low-throughput process. This study investigates the efficacy of a handheld near-infrared spectrometer device (NIRS) for the non-destructive, rapid prediction of these traits. The research methodology involved collecting spectral data from 2,236 cassava clones from 19 field trials in Brazil, using two sample types: fresh roots and mashed roots. Six spectral pre-processing methods and three machine learning algorithms—Partial Least Squares (PLS), Support Vector Machines (SVM), and Extreme Gradient Boosting (XGB)—were evaluated to optimize predictive models. Model performance was assessed using the coefficient of determination in calibration ($R_{C}$), the root mean squared error of calibration (${RMSE}_{C}$), and the Kappa index to quantify the consistency of clone selection. Results show that mashed samples consistently yielded superior predictive performance across all models. Specific preprocessing methods, such as Savitzky-Golay filtering combined with Standard Normal Variate (SG + SNV) and first-derivative transformations, significantly enhanced model accuracy. Among the algorithms, PLS demonstrated the best overall performance, with high predictive accuracy ($R_{C}$ >0.96) and low prediction errors (${RMSE}_{C}$<1.3 for DMCo), especially with mashed samples. High Kappa index values, consistently approaching 1.0, confirmed a good alignment between NIRS-based selection and traditional phenotypic methods. This study validates a portable spectrometer as a reliable and efficient tool for high-throughput phenotyping in cassava breeding programs. The findings confirm that portable NIRS devices, when used with optimal sample preparation (mashed roots) and robust modeling (PLS), can effectively yield good predictions for plant selection. This approach can significantly accelerate breeding cycles by enabling rapid, early-stage selection decisions, thereby overcoming a major bottleneck and contributing to a more efficient and sustainable genetic improvement of cassava.

## Introduction

Cassava (*Manihot esculenta* Crantz) is an important crop for food and economic security across many tropical regions, including Africa, Latin America, and Southeast Asia, due to its exceptional energy-use efficiency and remarkable adaptability to challenging environmental conditions [1,2]. In addition to its direct role in human diets, cassava roots are an increasingly valuable industrial raw material, with diverse and expanding applications in the food, chemical, cosmetic, energy, and biodegradable packaging sectors [2–5]. Chemically, cassava roots are rich in starch, comprising approximately 83% amylopectin and 17% amylose [6,7], and contain relatively low protein levels (0.7–1.3% fresh weight) [8]. Compared with other staples, cassava delivers about 40% more carbohydrates than rice and 25% more than maize, making it one of the most efficient calorie sources for both humans and livestock [9]. This productivity, combined with its unique chemical profile, grants cassava strategic importance not only as an affordable nutritional resource but also as a versatile raw material for diverse industrial applications. The combination of high carbohydrate yield, agronomic resilience, and strong potential for value-added processing has made cassava a priority target for genetic improvement programs. Such programs not only seek to increase root yield but also aim to enhance technological quality traits with direct implications for the entire value chain.

Among these traits, dry matter content (DMC) and starch content (StC) stand out as priorities due to their strong correlations with industrial processing yield, functional properties of roots, and consumer acceptance [10–12]. In industrial cassava production systems, grower payments are often determined by DMC yield [11], making it a critical indicator of both quality and profitability. Consequently, identifying genotypes with elevated DMC and StC is a fundamental step toward achieving consistent genetic gains. However, conventional phenotyping approaches for DMC and StC present significant limitations. Simple techniques such as weighing roots in air and subsequently in water [13] are easy to apply in the field but suffer from reduced accuracy due to environmental, genetic, and management variability [14–16]. Errors in DMC estimation can mislead genotype selection, delaying breeding cycles and reducing the efficiency of selection. Conversely, more accurate reference methods, such as oven-drying to constant weight or starch extraction [17] are time-consuming, require specialized infrastructure, and are poorly scalable. This hinders their application in early breeding stages, especially in programs with limited resources [18,19]. These challenges underscore the urgent need for high-throughput, portable, and cost-effective analytical tools capable of delivering precise, non-destructive, real-time measurements [20,21].

Near-infrared spectroscopy (NIRS) has emerged as one of the most promising tools to address these constraints, enabling rapid, simultaneous, and non-destructive chemical analyses in solid, liquid, and semi-solid materials, including agricultural products [22–24]. The technique is based on the excitation of specific molecular vibrations—such as C–H, O–H, N–H, and C = O bonds—allowing for the quantification of organic constituents through the interpretation of unique spectral signatures [25–28]. NIRS has been extensively applied for the estimation of key chemical parameters, including moisture, starch, protein, and lipid content, across diverse matrices, and has proven particularly valuable in plant breeding programs [27,29,30].

In cassava, NIRS has been successfully used to predict traits such as DMC [18,31,32], carotenoid content [31,32], pasting properties [33,34], starch content [35], cyanogenic potential [32,36], and cooking time [37,38]. These predictions have traditionally been achieved using benchtop spectrometers, devices designed for laboratory conditions with controlled temperature and lighting and high optical resolution [39,40]. For example, Nuwamanya et al. [41] achieved high predictive accuracies (0.96 for DMC and 0.94 for amylose) using the DS2500 benchtop spectrometer to evaluate 300 cassava clones in Uganda. Despite their analytical precision, benchtop spectrometers are expensive, lack portability, and require laboratory infrastructure. Even portable full-range spectrometers face practical barriers, including high costs, proprietary software, and dependence on internet connectivity, all of which limit their applicability in remote field environments [5].

Recent developments in compact and affordable NIRS devices, such as the SCiO molecular sensor, have significantly expanded opportunities for in-field spectroscopy, particularly in low-infrastructure settings. The SCiO is a portable, lightweight, key chain-sized device that connects via Bluetooth to a smartphone for spectrum acquisition and processing, enabling rapid, non-destructive, real-time measurements across diverse sample types. The device operates over a narrower spectral range (740–1070 nm) than conventional full-range spectrometers. Despite this limitation, it has shown promising performance in predicting quality traits in plant products. Mbanjo et al. [35], for example, evaluated 344 cassava clones from the International Institute of Tropical Agriculture (IITA) breeding program, comparing SCiO performance with two conventional full-range spectrometers: the ASD QualitySpec® Trek (350–2500 nm) and the FOSS XDS Rapid Content™ Analyzer (400–2490 nm). They reported prediction accuracies between 0.84 and 0.90 for SCiO, demonstrating that even with a reduced spectral range, the device can produce results comparable to laboratory-grade instruments. These features make SCiO highly attractive for breeding programs that require large-scale phenotyping and fast decision-making. However, its application to Brazilian cassava germplasm for the prediction of DMC and starch content remains limited. Systematic studies evaluating factors influencing SCiO performance, such as sample type, spectral preprocessing, and modeling algorithms, are scarce. It is therefore necessary to assess SCiO across different breeding populations, as these may differ in chemical composition and root structure. Such differences can arise from genetic variation among clones, as well as from environmental and management factors, including climate, soil, and agronomic practices, all of which can directly influence root traits and, consequently, the accuracy of SCiO measurements. This approach is essential to ensure reliable prediction models that are applicable to cassava breeding programs, especially given that the crop is highly heterozygous and genetically diverse.

Among these factors, sample type is one of the most critical, as it directly influences spectral quality and reproducibility. Processed (mashed) samples generally produce more homogeneous spectra with reduced physical interference, thereby enhancing predictive accuracy. In contrast, fresh, intact samples—while more practical and aligned with field phenotyping—tend to introduce greater spectral variability due to factors such as surface moisture, texture, and root heterogeneity [18,31,42,43]. This variability can significantly affect prediction robustness, especially when using spectrometers with limited spectral ranges like the SCiO, making empirical validation essential to determine their suitability in different contexts.

In addition to sample type, spectral preprocessing is fundamental for building robust calibration models. Proper preprocessing can highlight spectral regions associated with target compounds while minimizing noise, scattering effects, and other irrelevant sources of variation. Commonly applied techniques include multiplicative scatter correction (MSC), standard normal variate (SNV), Savitzky–Golay smoothing (SG), first derivative transformation, and detrending [31,35,38]. By carefully combining optimized sample preparation with appropriate preprocessing methods, researchers can maximize the informative content of spectra, thereby improving model accuracy and reliability. This integrated strategy ensures that SCiO can deliver consistent and meaningful predictions even in complex breeding populations and variable field conditions, ultimately supporting rapid, high-throughput phenotyping in practical breeding scenarios.

Finally, the choice of statistical modeling algorithm plays a decisive role in converting spectral data into reliable quantitative predictions. Partial least squares regression (PLS) is widely adopted for its robustness to multicollinearity among spectral variables [44]; however, as a linear technique, it may have limited ability to capture complex, nonlinear relationships. Machine learning methods such as Support Vector Machines (SVM) and Extreme Gradient Boosting (XGB) have therefore been explored as powerful alternatives [23,45]. The SVM model is effective in mapping data into high-dimensional spaces to identify optimal decision boundaries, while XGB, based on gradient-boosted decision trees, is recognized for its robustness to noise and ability to capture nonlinear interactions.

Despite the rapid technical progress in NIRS spectroscopy, uncertainties remain regarding the ability of portable devices to deliver accurate predictions across genetically diverse cassava germplasm and under varying sample preparation methods. Accordingly, the objectives of this study were to: (i) evaluate the predictive performance of the portable SCiO spectrometer for estimating DMC and StC in Brazilian cassava breeding and germplasm populations; (ii) compare calibration performance across three modeling algorithms (PLS, SVM, and XGB); and (iii) assess model accuracy for different root sample types (fresh and mashed), with the aim of providing practical guidelines for the adoption of portable NIRS technologies in cassava breeding programs.

## Materials and methods

### Plant material and field trials

This study evaluated 2,236 cassava clones from the breeding program of Embrapa Mandioca e Fruticultura, headquartered in Cruz das Almas, Bahia, Brazil (12°39′25″ S, 39°07′27″ W, 226 m altitude). The genetic materials were tested between 2018 and 2023 in 19 field trials, encompassing different stages of the breeding pipeline, including local varieties (germplasm – BAG), parental lines for crossing blocks (CB), seedling evaluation trials (SET), clonal evaluation trials (CET), preliminary yield trials (PYT), advanced yield trials (AYT), and uniform yield trials (UYT). The experiments were carried out across eight experimental stations distributed throughout the state of Bahia, representing different edaphoclimatic zones of the humid and sub-humid tropical region, with a climate classified as Aw/Am according to Köppen–Geiger [46]. The mean annual temperature is approximately 25 °C, with annual precipitation ranging from 900 to 1,300 mm.

### Experimental conditions and phenotyping

All trials were conducted under rainfed conditions, taking advantage of the natural rainfall regime between May and July. For SET, CB, CET, PYT, and AYT trials, a randomized complete block design with two or three replications was used. The BAG trials employed an augmented block design with ten checks per block. Planting was performed using 15–20 cm stem cuttings (5–7 buds) at a spacing of 0.90 m between rows and 0.80 m between plants. Basal fertilization consisted of 40 kg ha ⁻ ¹ of P₂O₅ at planting, followed by a topdressing application of 30 kg ha ⁻ ¹ of N and 45 kg ha ⁻ ¹ of K₂O at 50–60 days after planting, according to the recommendations of Souza et al. [47]. Harvest occurred 11–12 months after planting.

From each plot, 6–10 intact roots per clone (free of damage, pests, or diseases) were randomly selected, washed to remove soil, and trimmed to discard both ends. Selection criteria included root shape, typical cultivar size, and absence of pest or disease symptoms. The following traits were measured: Root dry matter content (DMCg, %) by weighing roots in air and then submerged in water, following Kawano et al. [13]; Oven-dry matter content (DMCo, %) of chopped and milled roots dried at 90 °C to constant weight, according to Carvalho et al. [48]; Starch content (StC, %) extracted from roots according to the protocols of Sánchez et al. [49] and Vasconcelos et al. [17].

### Spectral data collection and analysis

Spectral data were collected using near-infrared spectroscopy (NIRS) with a portable SCiO spectrometer (Consumer Physics Inc., Tel Aviv, Israel). This handheld device operates in diffuse reflectance mode over the spectral range of 740–1,070 nm (equivalent to 13,514–9,346 cm ⁻ ¹). The instrument contains 12 photodetectors, each equipped with a specific optical filter. The average spectral resolution is 13 cm ⁻ ¹, ranging from 9 cm ⁻ ¹ at longer wavelengths to 18 cm ⁻ ¹ at shorter wavelengths. The spectrometer connects to a smartphone via Bluetooth, using the SCiO^TM^ Lab app for spectrum acquisition, management, and storage. Each spectral scan lasted approximately 2 s, and data were transmitted and automatically stored on the SCiO server. To maintain a standardized 9 mm distance between the sensor and the sample, and to prevent ambient light interference, a plastic light shield provided by the manufacturer was used in all measurements.

For spectral acquisition, 15 roots per experimental plot were randomly selected, originating from three competitive plants (five healthy roots per plant). Roots were selected with diameters between 4 and 7 cm, washed under running water to remove debris, and manually peeled. From each root, two central sections, approximately 10 cm long were excised, with diameters determined by genotype characteristics. Spectral data were collected from two sample types (fresh and mashed) to enable comparison of predictive model performance across different physical states of the root. For fresh samples, spectra were obtained directly from the exposed surface of one central root section, with 47 spectral readings per plot to ensure robust representation. For mashed samples, the second central section of each root was chopped into ~3 × 10 mm pieces, further minced, and homogenized using a household food processor to obtain a uniform pulp. Approximately 8 g of the mashed sample were placed in quartz cuvettes and positioned over the spectrometer’s reading window, with four readings taken per plot.

### Spectral data preprocessing

To identify the most effective preprocessing approach for spectral analysis and ensure the reliability of spectral data, different combinations of pre-treatment methods were compared with raw data (without preprocessing), including: (i) first derivative (1st derivative), used to remove background and baseline drift effects [50,51], (ii) standard normal variate (SNV), and (iii) multiplicative scatter correction (MSC). The SNV and MSC preprocessing methods are designed to eliminate multiplicative scattering interferences in spectral signals. The difference between them is that SNV normalizes each spectrum individually by centering it to a mean of zero and scaling it to unit variance, thereby reducing multiplicative scatter effects, whereas MSC aligns each spectrum to a common reference, typically the mean spectrum of the dataset, through linear regression, correcting both baseline offset and slope [52]. Additionally, combinations of pre-treatments were evaluated: (i) first derivative combined with detrend (1st + DT), with DT used to remove baseline drift [53], (ii) first derivative combined with MSC (1st + MSC), and (iii) Savitzky-Golay (SG) combined with SNV (SG + SNV), configured using a third-order polynomial and an 11-point moving window. The first derivative emphasizes subtle spectral variations while reducing background noise by fitting local polynomial regressions to successive segments of the spectrum, effectively smoothing the data without distorting key features such as peaks and troughs [54]. To further improve baseline correction and suppress high-frequency noise, the SG filter was integrated with the gap-segment derivative method, which calculates the derivative between data points separated by a fixed interval (window size = 11, segment size = 7), enhancing sensitivity to minor spectral differences and improving signal clarity for downstream analysis. The SNV preprocessing was also applied to this configuration (SG + SNV). Each preprocessing method was tested to reduce instrumental noise, light-scattering effects, and systematic variations between samples while preserving the integrity of spectral features relevant for modeling. Comparing preprocessing strategies was essential to select the approach that best balanced noise reduction with the preservation of chemometric information, in line with the study’s predictive objectives. Therefore, a comparison was conducted between the raw spectral data (control group) and various spectral preprocessing methods (1st derivative, SNV, MSC, 1st + detrend, 1st + SNV, 1st + MSC, and SG + SNV), based on two key statistical parameters: predictive accuracy (${\text{R}}_{\text{pt}}$) and root mean squared error of the pre-treatment (${\text{RMSE}}_{\text{pt}}$). These six preprocessing strategies were implemented using the prospectr package [55]. 

To minimize bias from extreme values and ensure data quality, outliers were identified and removed based on the interquartile range (IQR), with exclusion defined as any sample falling beyond 1.5 times the IQR [56]. This procedure was applied to both raw and pre-processed spectra, allowing for the removal of aberrant data points potentially caused by measurement errors, instrumental variations, or anomalous sample behavior. Eliminating these points helped reduce noise and improve the robustness of predictive models. After preprocessing and spectral data cleaning, the information was organized into a predictor matrix (X), containing spectral variables (absorbance), and a response vector (Y), comprising the observed values of root quality attributes: starch content (StC), oven-dried matter (DMCo), and gravimetric dry matter (DMCg). For model development and evaluation, the dataset was randomly split into two subsets: 80% of the data were used for model calibration (training set), and the remaining 20% were reserved for external validation. This partitioning enabled independent assessment of model performance on unseen data, providing a realistic estimate of their generalization ability.

### Optimization of spectral preprocessing

To determine the most effective spectral preprocessing strategy for predicting cassava root quality traits, partial least squares (PLS) regression model was fitted to both the raw spectral data and to each of the evaluated preprocessing methods (1st derivative, SNV, MSC, 1st derivative + DT, 1st derivative + MSC, and SG + SNV). Model calibration followed a repeated cross-validation protocol consisting of five repetitions of 5-fold cross-validation, applied exclusively to the calibration set. Prior to final model fitting, an outlier screening step was conducted using residuals from an initial model iteration. The 20% of calibration samples with the largest residual deviations were removed to minimize the influence of extreme values and enhance model robustness. Predictive performance was then evaluated based on Pearson’s correlation coefficient (${\text{R}}_{\text{pt}}$) between observed and predicted values, and the root mean squared error of prediction (${\text{RMSE}}_{\text{pt}}$). Models were developed separately for fresh and mashed samples. The most suitable preprocessing method was defined as the one consistently yielding the highest ${\text{R}}_{\text{pt}}$ and lowest ${\text{RMSE}}_{\text{pt}}$ across both sample types, thus demonstrating stability and predictive strength regardless of the physical state of the root.

### Spectral calibration models

To compare predictive performance, three regression algorithms were evaluated for estimating cassava root quality traits. The first was partial least squares regression (PLS), which projects both predictor (X) and response (Y) matrices into a lower-dimensional latent space. These latent variables, or PLS components, are constructed to maximize the covariance between X and Y, making PLS particularly effective for high-dimensional, collinear datasets [57,58]. The second was the support vector machine (SVM), a supervised learning algorithm that identifies an optimal hyperplane to separate data points from different classes. By mapping input variables into a higher-dimensional space through kernel functions, SVM can capture complex, non-linear relationships while maintaining strong generalization performance [59,60]. The third was eXtreme Gradient Boosting (XGB), a highly efficient and scalable implementation of the gradient-boosted decision tree (GBDT) algorithm [61]. Unlike traditional ensemble methods such as random forests [62]. The XGB model incorporates explicit regularization to reduce overfitting, improve generalization, and accelerate computation through optimized routines [63]. Based on prior performance assessments, the boosting process was configured with 200 iterations (*nrounds*) and a maximum tree depth (*max_depth*) of 5 to control complexity. The learning rate (*eta*) was set to 0.3 to balance convergence speed and predictive accuracy. Additional regularization included a minimum loss reduction threshold (*gamma* = 5) required before allowing further tree splits. To increase diversity among trees and reduce overfitting, each iteration randomly sampled 70% of the training instances (*subsample* = 0.7) and 70% of the predictor variables (*colsample_bytree* = 0.7). A minimum child weight of 1 was applied to allow flexible branching. All modeling was conducted in R software (version 4.4.2) [64] using the caret package [65].

### Cross-validation and model evaluation

Model training employed repeated 5-fold cross-validation with five repetitions, applied exclusively to the training set (80% of the dataset). Outliers within the training data were identified from residuals in an initial modeling run, and the 20% of samples with the largest residuals were removed. Models were then refitted using the refined dataset. Performance was assessed using several metrics: Pearson’s correlation coefficient (R) between predicted and observed values, the coefficient of determination ($R^{2}$), bias, and the root mean squared error (RMSE) for both cross-validation and external validation sets. Additionally, two scale-independent performance ratios were calculated: the ratio of performance to deviation (RPD), defined as: $RPD=\frac{\sigma }{RMSE}$, where σ is the standard deviation (SD) of the reference or predicted values [31,66] and the ratio of performance to interquartile range (RPIQ) as follow: $RPIQ=\frac{IQR}{RMSE}$; with IQR being the interquartile distance (Q3−Q1) [67,68]. This multi-metric evaluation allowed for a robust comparison of modeling strategies across preprocessing methods and sample types.

### Concordance between phenotypic selection and predictive selection

Cohen’s unweighted kappa statistic [69] was used to quantify agreement between phenotypic rankings and model-based predictions, as well as to compare the consistency of selection decisions across predictive models. To evaluate clone performance across environments, a combined analysis of variance was conducted using the following mixed linear model:


$$Y_{ijlk}=\mu +g_{i}+\beta_{j}+r_{l(j)}+\rho_{k(l)}+{g\beta }_{ij}+\varepsilon_{ijlk}$$ (1)

where $Y_{ijlk}$ is the observed value of the *i*^th^ clone in the *j*^th^ environment (year × location combination), within the *l*^th^ replication and the *k*^th^ b*l*ock; μ is the overall mean; $g_{i}$ = random effect of the *i*^th^ clone ($\sim N(0,I{\hat{\sigma }}_{g}^{2})$); $\beta_{j}$: is the fixed effect of the environment; $r_{l(j)}$ is the random effect of replication nested within environment ($\sim N(0,I{\hat{\sigma }}_{r}^{2}))$; $\rho_{k(l)}$ is the random effect of block nested within replication and environment ($\sim N(0,I{\hat{\sigma }}_{k}^{2}))$; ${g\beta }_{ij}$ is the random genotype × environment interaction ($\sim N(0,I{\hat{\sigma }}_{ge}^{2}))$; $\varepsilon_{ijlk}$ is the residual error ($\sim N(0,I{\hat{\sigma }}_{e}^{2}))$. Given the unbalanced number of clones per trial and the variation in experimental designs across locations, both best linear unbiased predictions (BLUPs) and deregressed BLUPs (dBLUPs) were calculated for each trait. Deregression followed Garrick et al. [70]:


$$dBLUP=\frac{BLUP}{1-\frac{PEV}{{\hat{\sigma }}_{g}^{2}}}$$ (2)

where PEV is the prediction error variance associated with the BLUP, and ${\hat{\sigma }}_{g}^{2}$ is the genotypic variance component. Analyses were performed in R software (version 4.4.2) [64] using the LMMsolver package [71].

Based on dBLUPs, confusion matrices were constructed to identify the top 20% of clones for each trait. This allowed us to determine whether the same genotypes selected using phenotypic data were also selected using model predictions—thereby assessing the practical relevance of the models for breeding decisions. The kappa index was calculated as:


$$Kappa=\frac{p_{o}+p_{e}}{1-p_{e}}$$ (3)

where $p_{o}$ is the proportion of actual agreement between classification methods, and $p_{e}$ is the agreement expected by chance. The kappa coefficient thus reflects the extent to which model-based selection surpasses random selection. Values near 1 indicate strong agreement between selection approaches and, consequently, high reliability of the models in correctly identifying desirable genotypes [72].

## Results

### Selection of spectral preprocessing methods

**Fig 1:**
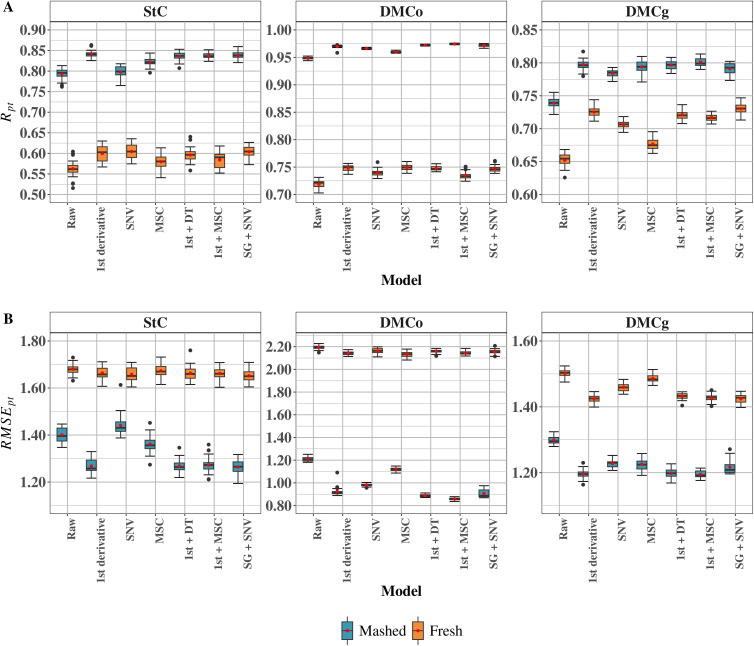
Near-infrared (NIR) spectra processed using different spectral pre-treatment methods for two sample types (mashed and fresh). (A) Cross-validation accuracy (${\text{R}}_{\text{pt}}$); (B) Root mean squared error (${\text{RMSE}}_{\text{pt}}$). Raw: unprocessed data; 1st derivative: first derivative; SNV: standard normal variate; MSC: multiplicative scatter correction; 1st + DT: first derivative plus detrending; 1st + MSC: first derivative plus MSC; SG + SNV: Savitzky–Golay smoothing plus SNV. StC: starch content; DMCo: dry matter content determined by the oven-drying method; DMCg: dry matter content determined by the gravimetric method.

A comparison was performed between raw spectral data and various preprocessing methods, evaluating predictive accuracy (${\text{R}}_{\text{pt}}$) and root mean squared error (${\text{RMSE}}_{\text{pt}}$) (Fig 1). The results showed that preprocessing type influenced prediction quality, with effects varying according to sample type (fresh vs. mashed) and target trait. Overall, even without preprocessing, mashed samples achieved higher ${\text{R}}_{\text{pt}}$ values compared to fresh samples, particularly for starch content (StC) and dry matter content determined by oven-drying (DMCo) (Fig 1A).

For StC prediction in mashed samples, spectral pre-treatment had a high impact on model performance. Using raw spectral data resulted in ${\text{R}}_{\text{pt}}$ = 0.79 and ${\text{RMSE}}_{\text{pt}}$ = 1.40. Preprocessing enhanced predictive accuracy, with the 1st derivative, 1st derivative + DT, 1st derivative + MSC, and SG + SNV methods all achieving ${\text{R}}_{\text{pt}}$ = 0.84 and ${\text{RMSE}}_{\text{pt}}$ values ranging from 1.26 to 1.27 (Fig 1). Among these, SG + SNV yielded the lowest ${\text{RMSE}}_{\text{pt}}$ (1.26), indicating superior prediction precision despite similar accuracy levels across methods. In fresh samples, predictive accuracy was consistently lower than in mashed samples across all pre-treatments (Fig 1A). The ${\text{R}}_{\text{pt}}$ values ranged from 0.56 for raw data to 0.60 for the 1st derivative, SNV, 1st derivative + DT, and SG + SNV, while ${\text{RMSE}}_{\text{pt}}$ values ranged from 1.65 to 1.68 (Fig 1). Once again, SG + SNV produced the lowest prediction error (1.65), even though accuracy values among pre-treatments were closely aligned.

Similarly, for DMCg prediction in mashed samples, spectral pre-treatments also had a significant impact on model performance. Using raw spectral data yielded ${\text{R}}_{\text{pt}}$ = 0.74 and ${\text{RMSE}}_{\text{pt}}$ = 1.30 (Fig 1). Preprocessing improved results, with the 1st derivative, 1st derivative + DT, and 1st derivative + MSC methods achieving ${\text{R}}_{\text{pt}}$ = 0.80 and ${\text{RMSE}}_{\text{pt}}$ = 1.20. Although SG + SNV produced slightly lower accuracy (${\text{R}}_{\text{pt}}$ = 0.79) and a marginally higher ${\text{RMSE}}_{\text{pt}}$ (1.22) than these top-performing methods, its performance still exceeded that obtained with unprocessed data. Isolated application of SNV and MSC also yielded moderate improvements in predictive accuracy (${\text{R}}_{\text{pt}}$ = 0.78 and 0.79, respectively), albeit with slightly higher errors (${\text{RMSE}}_{\text{pt}}$ = 1.23). Compared with mashed samples, fresh samples for DMCg prediction displayed a pronounced drop in performance, with lower ${\text{R}}_{\text{pt}}$ values and higher ${\text{RMSE}}_{\text{pt}}$ values (Fig 1). The model calibrated with raw data reached ${\text{R}}_{\text{pt}}$ = 0.65 and ${\text{RMSE}}_{\text{pt}}$ = 1.50, both notably lower in accuracy and higher in error than those for mashed samples (0.74 and 1.30, respectively). After spectral preprocessing, accuracy improved, particularly with the 1st derivative and SG + SNV methods, both achieving ${\text{R}}_{\text{pt}}$ = 0.73 and ${\text{RMSE}}_{\text{pt}}$ = 1.42 (Fig 1). Calibration with 1st derivative + DT, 1st derivative + MSC, and SNV also improved accuracy compared to raw data, with ${\text{R}}_{\text{pt}}$ ranging from 0.71 to 0.72 and ${\text{RMSE}}_{\text{pt}}$ from 1.43 to 1.46. By contrast, the isolated use of MSC yielded more limited performance relative to other pre-treatments (${\text{R}}_{\text{pt}}$ = 0.68; ${\text{RMSE}}_{\text{pt}}$ = 1.49) (Fig 1).

For DMCo prediction in mashed samples, the use of different spectral pre-treatments yielded higher predictive performance compared with the other evaluated traits (Fig 1). Even without preprocessing, raw spectral data achieved a high ${\text{R}}_{\text{pt}}$ of 0.95 and an ${\text{RMSE}}_{\text{pt}}$ of 1.21. Applying spectral preprocessing further improved results, with better performance from 1st derivative, SNV, SG + SNV, 1st derivative + DT, and 1st derivative + MSC, which achieved ${\text{R}}_{\text{pt}}$ values of 0.96–0.97 and ${\text{RMSE}}_{\text{pt}}$ values of 0.86–0.98 (Fig 1). In contrast, although the isolated use of MSC resulted in a relatively high accuracy (${\text{R}}_{\text{pt}}$ = 0.96), it produced the largest ${\text{RMSE}}_{\text{pt}}$ (1.12) among all tested pre-treatments (Fig 1). For fresh samples, predictive models for DMCo performed worse than those for mashed samples, with consistently lower accuracy and higher prediction errors. The model calibrated with raw data yielded ${\text{R}}_{\text{pt}}$ = 0.72 and ${\text{RMSE}}_{\text{pt}}$ = 2.19. Pre-treatments such as SNV and 1st derivative + MSC offered slight improvements over raw data (${\text{R}}_{\text{pt}}$ = 0.74 and ${\text{RMSE}}_{\text{pt}}$ = 2.14 for both). However, the use of 1st derivative, MSC, 1st derivative + DT, and SG + SNV achieved marginally higher accuracy (${\text{R}}_{\text{pt}}$ = 0.75) and ${\text{RMSE}}_{\text{pt}}$ values ranging from 2.13 to 2.16 (Fig 1).

### Assessment of prediction accuracy across models

**Fig 2:**
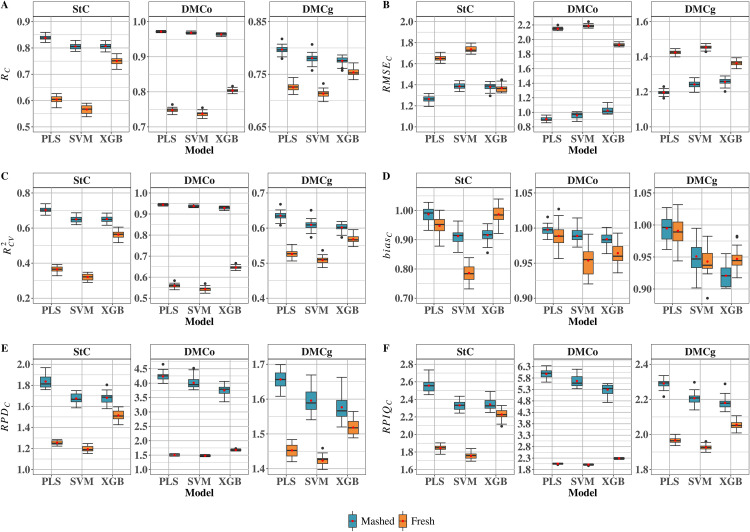
Calibration performance based on near-infrared (NIR) spectra collected using pocket-sized SCiO molecular sensor. Performance metrics are shown for different calibration models and cassava root sample types (mashed and fresh). (A) Prediction accuracy of cross-validation ($R_{C}$); (B) Root mean square error of cross-validation (${RMSE}_{C}$); (C) Coefficient of determination of cross-validation ($R_{CV}^{2}$); (D) Calibration bias (${bias}_{C}$) (E) ratio of performance to deviation (${RPD}_{C}$) and (F) ratio of performance to inter quartile range (${RPIQ}_{C}$). StC: starch content; DMCo: dry matter content determined by the oven-drying method; DMCg: dry matter content determined by the gravimetric method.

The performance and accuracy of PLS, XGB, and SVM models were evaluated for different sample types (mashed and fresh). This assessment was based on a comprehensive set of statistical metrics: prediction accuracy of cross-validation ($R_{C}$), root mean squared error of cross-validation (${RMSE}_{C}$), coefficient of determination of cross-validation ($R_{CV}^{2}$), calibration bias (${bias}_{C}$), ratio of performance to deviation (${RPD}_{C}$), and ratio of performance to interquartile range (${RPIQ}_{C}$) (Fig 2). Overall, models calibrated with mashed samples demonstrated better predictive performance compared to those calibrated with fresh samples, as evidenced by higher $R_{C}$ and lower ${RMSE}_{C}$ values.

For StC in mashed samples, all three models (PLS, SVM, and XGB) demonstrated good predictive ability, with metrics indicating adequate fit and generalization (Fig 2). The PLS model achieved the best overall performance, with $R_{C}$ = 0.84 and $R_{CV}^{2}$ = 0.70. It also showed the lowest ${RMSE}_{C}$ (1.26) and ${bias}_{C}$ (0.99), alongside the highest ${RPD}_{C}$ (2.56) and ${RPIQ}_{C}$ (1.84). The SVM and XGB models displayed similar performance, with slightly lower $R_{C}$ (0.81) and $R_{CV}^{2}$ (0.65), slightly higher ${RMSE}_{C}$ values (1.39 for SVM and 1.38 for XGB), and biases of 0.91 and 0.92, respectively. Their ${RPD}_{C}$ values were 2.33 (SVM) and 2.34 (XGB), while ${RPIQ}_{C}$ was 1.68 for both. For fresh samples, StC predictions varied among models. The XGB model performed best, with $R_{C}$ = 0.75, $R_{CV}^{2}$ = 0.56, ${RMSE}_{C}$ = 1.37, ${bias}_{C}$ = 0.99, ${RPD}_{C}$ = 2.23, and ${RPIQ}_{C}$ = 1.51. The PLS model showed slightly lower performance ($R_{C}$ = 0.60, $R_{CV}^{2}$ = 0.36, ${RMSE}_{C}$ = 1.65, ${bias}_{C}$ = 0.95, ${RPD}_{C}$ = 1.84, and ${RPIQ}_{C}$ = 1.25), while SVM had the lowest accuracy, with $R_{C}$ = 0.57, $R_{CV}^{2}$ = 0.32, ${RMSE}_{C}$ = 1.74, ${bias}_{C}$ = 0.79, ${RPD}_{C}$ = 1.75, and ${RPIQ}_{C}$ = 1.19 (Fig 2).

For DMCo using mashed samples, all models exhibited high predictive performance, with $R_{C}$ ≥ 0.96 and $R_{CV}^{2}$> 0.93. The PLS model stood out, achieving the lowest ${RMSE}_{C}$ (0.91), highest ${RPD}_{C}$ (5.98), and ${bias}_{C}$ of 1.0. SVM ($R_{C}$ = 0.97; $R_{CV}^{2}$ = 0.94) and XGB ($R_{C}$ = 0.96; $R_{CV}^{2}$ = 0.93) also performed consistently, with ${RMSE}_{C}$ of 0.96 and 1.02, respectively. All models showed high ${RPD}_{C}$ and ${RPIQ}_{C}$ values (>3.70 and >5.20, respectively), with the highest metrics observed for PLS (${RPD}_{C}$ = 4.23; ${RPIQ}_{C}$ = 4.23) and SVM (${RPD}_{C}$ = 4.01; ${RPIQ}_{C}$ = 4.01). In fresh samples, predictive performance for DMCo was moderate. The XGB model achieved the highest $R_{C}$ (0.80) and $R_{CV}^{2}$ (0.65), the lowest ${RMSE}_{C}$ (1.93), and the highest ${RPD}_{C}$ (2.29) and ${RPIQ}_{C}$ (1.68), with ${bias}_{C}$ close to one (0.96). PLS ($R_{C}$ = 0.75; $R_{CV}^{2}$ = 0.56) and SVM ($R_{C}$ = 0.74; $R_{CV}^{2}$ = 0.54) showed slightly lower performance, although biases were also near one (Fig 2).

For DMCg using mashed samples, all three models demonstrated comparable performance with minor differences. The PLS model achieved the lowest ${RMSE}_{C}$ (1.20), highest $R_{C}$ (0.80) and $R_{CV}^{2}$ (0.64), and the best ${RPD}_{C}$ (2.29) and ${RPIQ}_{C}$ (1.66), with ${bias}_{C}$ close to one (0.99). The SVM and XGB models performed slightly worse: SVM had ${RMSE}_{C}$ = 1.24, $R_{C}$ = 0.78, $R_{CV}^{2}$ = 0.61, ${RPD}_{C}$ = 2.21, ${RPIQ}_{C}$ = 1.60, and ${bias}_{C}$ = 0.95, while XGB had ${RMSE}_{C}$ = 1.26, $R_{C}$ = 0.78, $R_{CV}^{2}$ = 0.60, ${RPD}_{C}$ = 2.18, ${RPIQ}_{C}$ = 1.58, and ${bias}_{C}$ = 0.92. For DMCg predictions in fresh samples, ${RMSE}_{C}$ ranged from 1.36 (XGB) to 1.45 (SVM), with PLS at 1.42. $R_{C}$ values varied slightly (PLS = 0.73, SVM = 0.71, XGB = 0.75), while $R_{CV}^{2}$ ranged from 0.51 (SVM) to 0.57 (XGB). Overall, XGB showed superior performance. The ${RPD}_{C}$ values ranged from 1.93 (SVM) to 2.06 (XGB), and ${RPIQ}_{C}$ ranged from 1.42 (SVM) to 1.52 (XGB). Bias values remained close to 1 for all models (PLS = 0.99, SVM = 0.94, XGB = 0.95).

### External validation of prediction models

#### External validation models for different sample types.

**Fig 3:**
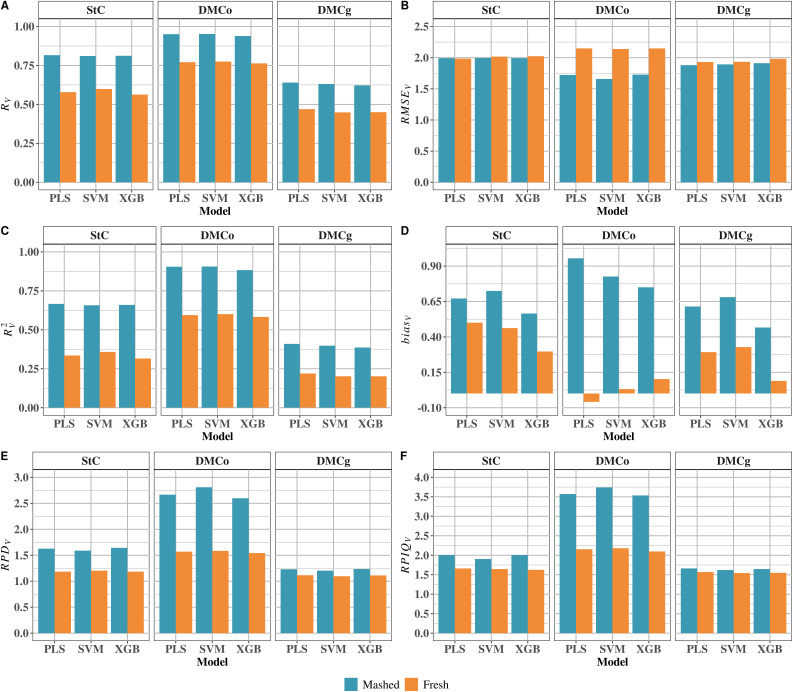
External validation performance of calibration models based on near-infrared (NIR) spectra collected using pocket-sized SCiO molecular sensor. (A) predictive accuracy ($R_{v}$), (B) root mean squared error of prediction (${RMSE}_{v}$), (C) coefficient of determination for prediction ($R_{v}^{2}$), (D) prediction bias (${bias}_{v}$), (E) ratio of performance to deviation (${RPD}_{V}$) and (F) ratio of performance to inter quartile range (${RPIQ}_{V}$), across different sample types (mashed and fresh) and target traits. Evaluated traits include starch content (StC), dry matter content by the oven-drying method (DMCo), and dry matter content by the gravimetric method (DMCg).

Predictive performance was consistently higher for mashed samples compared to fresh samples across all traits and models (Fig 3). For DMCg, PLS, SVM, and XGB models all achieved better predictive accuracy with mashed samples. The ${RMSE}_{v}$ values were slightly lower for mashed samples (1.88, 1.89, and 1.91 for PLS, SVM, and XGB, respectively) than for fresh samples (1.93, 1.93, and 1.98) (Fig 3B). Correspondingly, $R_{v}^{2}$ increased for mashed samples: PLS improved from 0.22 (fresh) to 0.41 (mashed), SVM from 0.20 to 0.40, and XGB from 0.20 to 0.39 (Fig 3C). Similar trends were observed for $R_{v}$, ${RPIQ}_{v}$, and ${RPD}_{v}$, with PLS achieving the highest values parameters for mashed samples ($R_{v}$ = 0.64, ${RPIQ}_{V}$ = 1.66, and ${RPD}_{V}$ = 1.23) (Fig 3). ${bias}_{v}$ was higher for mashed samples (0.47–0.68) than for fresh samples (0.09–0.33), indicating a slight tendency for overestimation.

For DMCo, differences between sample types were more pronounced. The SVM model based on mashed samples showed the lowest ${RMSE}_{V}$ (1.66) and highest $R_{V}^{2}$ (0.91), followed closely by XGB (${RMSE}_{V}$ = 1.73; $R_{V}^{2}$ = 0.88) and PLS (${RMSE}_{V}$ = 1.72; $R_{V}^{2}$ = 0.90) (Fig 3). The $R_{v}$ values were slightly higher for mashed samples (0.94–0.95) than fresh samples (0.76–0.78) (Fig 3A). Similarly, ${RPIQ}_{V}$ and ${RPD}_{V}$ were higher for mashed samples (up to 3.74 and 2.81, respectively), reflecting greater predictive robustness. ${bias}_{v}$ values were also slightly higher for mashed samples (0.75–0.96) compared with fresh samples (–0.06 to 0.10), indicating minor overestimation despite improved accuracy (Fig 3D).

For StC, predictive performance was again superior for mashed samples. The $R_{V}^{2}$ values for PLS, SVM, and XGB were 0.67, 0.66, and 0.66, respectively, compared with 0.34, 0.36, and 0.32 for fresh samples (Fig 3A). Predictive accuracy ($R_{v}$) was higher for mashed samples (0.81–0.82) than for fresh samples (0.56–0.60). ${RPIQ}_{V}$ and ${RPD}_{V}$ values were also greater for mashed samples (maximum 2.01 and 1.64) compared to fresh samples (1.66 and 1.21). ${bias}_{v}$ was higher for mashed samples (0.57–0.72) than fresh samples (0.30–0.50), reflecting a minor overestimation trend.

#### Starch Content (StC).

In external validation using mashed samples, all three models (PLS, SVM and XGB) showed similar ${RMSE}_{V}$ (1.99). $R_{v}$ was slightly higher for PLS (0.82) than SVM and XGB (0.81). The $R_{V}^{2}$ was 0.67 for PLS and 0.66 for SVM and XGB, indicating comparable predictive performance (Fig 3C). The ${RPD}_{V}$ ranged from 1.59 (SVM) to 1.64 (XGB), and ${RPIQ}_{V}$ from 1.90 (SVM) to 2.01 (PLS), reflecting moderate-to-good predictive ability (Fig 3E and 3F). The ${bias}_{v}$ varied among models, highest for SVM (0.72), followed by PLS (0.67) and XGB (0.57). For fresh samples, predictive performance was lower. The ${RMSE}_{V}$ was lowest for PLS (1.98) and slightly higher for SVM and XGB (2.02). The $R_{v}$ values ranged from 0.56 (XGB) to 0.60 (SVM), and $R_{V}^{2}$ from 0.32 (XGB) to 0.36 (SVM). The ${RPD}_{V}$ ranged from 1.18 (PLS) to 1.21 (SVM), while ${RPIQ}_{V}$ ranged from 1.63 (XGB) to 1.66 (PLS). The ${bias}_{v}$ varied from 0.30 (XGB) to 0.50 (PLS), with SVM intermediate (0.46).

#### Dry matter content by oven-drying method (DMCo).

For mashed samples, all models performed well. The PLS and SVM models achieved $R_{v}$ = 0.95, but SVM had the lowest ${RMSE}_{V}$ (1.66) and moderate ${bias}_{v}$ (0.83) (Fig 3). The SVM model also had high $R_{V}^{2}$ (0.91), ${RPIQ}_{V}$ (3.74), and ${RPD}_{V}$ (2.81). The PLS model had slightly higher ${RMSE}_{V}$ (1.72) but similar $R_{v}$ (0.95), $R_{V}^{2}$ (0.90), ${RPD}_{V}$ (2.67), and ${RPIQ}_{V}$ (3.57), with the highest ${bias}_{v}$ (0.96). The XGB model had slightly lower performance: ${RMSE}_{V}$ = 1.73, $R_{v}$ = 0.94, $R_{V}^{2}$ = 0.88, ${RPIQ}_{V}$= 3.54, ${RPD}_{V}$ = 2.60, and lowest ${bias}_{v}$ (0.75). For fresh samples, predictive performance decreased. The SVM model was slightly superior (${RMSE}_{V}$ = 2.14, ${bias}_{v}$≈ 0, $R_{v}$ = 0.78, and $R_{V}^{2}$ = 0.60), followed closely by PLS (${RMSE}_{V}$ = 2.15, $R_{v}$ = 0.77, $R_{V}^{2}$ = 0.60, and ${bias}_{v}$ = –0.06). The XGB model performed slightly worse (${RMSE}_{V}$ = 2.15, ${bias}_{v}$ = 0.10, $R_{v}$ = 0.76, and $R_{V}^{2}$ = 0.58). ${RPIQ}_{V}$ ranged from 2.10 (XGB) to 2.18 (SVM), and ${RPD}_{V}$ from 1.54 (XGB) to 1.58 (SVM).

#### Dry matter content by gravimetric method (DMCg).

For mashed samples, PLS, SVM, and XGB had comparable performance. The PLS model achieved $R_{v}$ = 0.64, $R_{V}^{2}$ = 0.41, ${RMSE}_{V}$ = 1.88, and ${bias}_{v}$ = 0.61. The SVM model was similar ($R_{v}$ = 0.63, $R_{V}^{2}$ = 0.40, ${RMSE}_{V}$ = 1.89, ${bias}_{v}$ = 0.68), while XGB performed slightly worse ($R_{v}$ = 0.62, $R_{V}^{2}$ = 0.39, ${RMSE}_{V}$ = 1.91, ${bias}_{v}$ = 0.47). The ${RPIQ}_{V}$ ranged from 1.62 (SVM) to 1.66 (PLS), and ${RPD}_{V}$ from 1.20 (SVM) to 1.24 (XGB). For fresh samples, all models showed lower performance. The PLS model had the highest $R_{v}$ and $R_{V}^{2}$ (0.47 and 0.22), with ${RMSE}_{V}$ = 1.93 and ${bias}_{v}$ = 0.29. The SVM and XGB models had similar $R_{v}$ and $R_{V}^{2}$ (0.45 and 0.20), but differed in ${RMSE}_{V}$ and ${bias}_{v}$: SVM ${RMSE}_{V}$ = 1.93, ${bias}_{v}$ = 0.33; XGB ${RMSE}_{V}$ = 1.98, ${bias}_{v}$ = 0.09. ${RPIQ}_{V}$ and ${RPD}_{V}$ differed minimally: PLS highest (1.57 and 1.12), followed by XGB (1.55 and 1.11) and SVM (1.54 and 1.09) (Fig 3).

### Agreement among models in cassava clone selection

**Fig 4:**
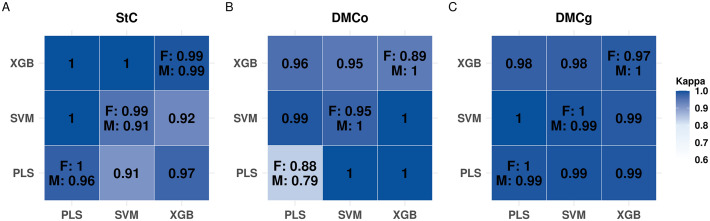
Heatmap of classification agreement (Cohen’s kappa) for cassava clone selection across different sample types. Evaluated traits include: (A) starch content (StC), (B) dry matter content by oven-drying (DMCo), and (C) dry matter content by the gravimetric method (DMCg). Each matrix shows pairwise agreement among prediction models: Partial Least Squares (PLS), Support Vector Machine (SVM), and Extreme Gradient Boosting (XGB). Lower-triangle values correspond to mashed samples, and upper-triangle values correspond to fresh samples. Diagonal cells display self-agreement for each model, with separate values for fresh (F) and mashed (M) samples. Color intensity reflects kappa values from 0.6 (light) to 1.0 (dark), representing agreement strength.

For the selection of the top 20% of clones based on StC in mashed samples, Cohen’s kappa values indicated a high level of agreement across all evaluated predictive models (Fig 4). For StC predictions in mashed samples, the XGB model showed the highest kappa (0.99), followed by PLS (0.96) and SVM (0.91), demonstrating that all algorithms effectively classified the majority of samples. Pairwise comparisons also revealed strong concordance in prediction patterns, with kappa values of 0.91 for PLS–SVM, 0.97 for PLS–XGB, and 0.92 for SVM–XGB, confirming consistency across different modeling approaches (Fig 4A).

Similarly, for StC in fresh samples, the models exhibited near-perfect agreement (Fig 4A). The PLS model achieved perfect concordance between predicted and observed values (kappa = 1.00), while SVM and XGB showed slightly lower but still very high agreement (kappa = 0.99 for both). Pairwise comparisons among the models revealed complete agreement in predictions, with PLS–SVM, PLS–XGB, and SVM–XGB all achieving kappa = 1.00, indicating robust and reliable performance across algorithms.

For DMCo in fresh and mashed samples, strong agreement between predicted and observed values was found (Fig 4B). Intra-model analysis showed high kappa values for all models. For fresh samples, the SVM model achieved a kappa of 0.95, while XGB and PLS presented kappa values of 0.89 and 0.88, respectively. For mashed samples, XGB and SVM showed perfect agreement (kappa = 1.00), whereas the PLS model showed slightly lower agreement (kappa = 0.79). Pairwise comparisons among the models for mashed samples revealed complete agreement for PLS–SVM, PLS–XGB, and SVM–XGB (kappa = 1.00). Similarly, kappa values for fresh samples also showed high concordance in prediction patterns, with kappa values of 0.99 for PLS–SVM, 0.96 for PLS–XGB, and 0.95 for SVM–XGB.

Comparable patterns were observed for dry matter content determined by the gravimetric method (DMCg) in mashed samples, with predicted and observed values showing high concordance, reflecting the robustness of the models (Fig 4C). Intra-model analysis revealed kappa values of 0.99 for both PLS and SVM, whereas XGB achieved perfect agreement (kappa = 1.00). Inter-model comparisons also showed consistently high kappa values (0.99 for PLS–SVM, PLS–XGB, and SVM–XGB), indicating stability and reliability across different modeling approaches.

For DMCg in fresh samples, kappa values remained high, both within and between models (Fig 4C). The PLS and SVM models exhibited perfect concordance (kappa = 1.00), while XGB maintained a slightly lower, yet still high, kappa of 0.97. Pairwise comparisons among models indicated near-complete agreement, with kappa values of 1.00 for PLS–SVM and 0.98 for both PLS–XGB and SVM–XGB, highlighting the strong consistency of predictive outcomes across methods.

## Discussion

### Importance of spectral pretreatment selection

The selection of an appropriate spectral pretreatment is a key factor determining the predictive performance of NIRS-based models, as the robustness of predictive models relies heavily on the quality of the spectral data used [73–75]. Raw spectra often contain noise and redundant information that can compromise calibration accuracy. Such noise may arise from instrumental factors, environmental conditions, variations in optical path length, and light scattering effects [74,75]. Therefore, applying pretreatments is essential to attenuate these interferences and remove irrelevant spectral information [73].

The impact of various preprocessing strategies (1st derivative, SNV, MSC, 1st + DT, 1st + SNV, 1st + MSC and SG + SNV) varied depending on both the sample type (mashed or fresh) and the phenotypic trait assessed (StC, DMCg, DMCo), as reflected in ${\text{R}}_{\text{pt}}$ and ${\text{RMSE}}_{\text{pt}}$ (Fig 1). Overall, mashed samples consistently outperformed fresh samples, likely due to their greater physical homogeneity, which reduces spectral variability arising from uneven distribution of compounds such as starch, dry matter, and carotenoids along the root, from the core to the peel and from apical to basal ends [76].

Regardless of the trait or sample type, pretreatments based on 1st derivative, 1st derivative + DT e SG + SNV consistently achieved superior performance across nearly all traits and sample types. However, the most effective pretreatment differed depending on the target trait, indicating that no single pretreatment is universally optimal. This variation likely reflects differences in chemical composition, sample physical properties, and analytical methods used for phenotypic assessment. For StC and DMCo, the combination of SG + SNV achieved the highest predictive performance among all pretreatments. In mashed samples, ${\text{R}}_{\text{pt}}$ values were 0.84 for StC and 0.97 for DMCo, with corresponding ${\text{RMSE}}_{\text{pt}}$ values of 1.26 and 0.91, respectively. While performance was lower in fresh samples, the SG + SNV approach still outperformed other pretreatments, yielding ${\text{R}}_{\text{pt}}$ of 0.60 (${\text{RMSE}}_{\text{pt}}$ = 1.65) for StC and 0.75 (${\text{RMSE}}_{\text{pt}}$ = 2.15) for DMCo (Fig 1). The Savitzky–Golay filter is widely recognized for its efficiency in spectral smoothing due to its computational simplicity, flexibility in applying derivatives of different orders, minimal parameter requirements, and ability to preserve the shape and intensity of spectral bands [77–80]. The SNV filter, in turn, removes multiplicative interferences associated with light scattering and particle size variability [81,82] making the combination particularly effective for heterogeneous plant matrices such as cassava roots [83,84]. Additionally, the predominance of C–H and O–H functional groups in compounds like starch and total solids may enhance the pretreatment’s effectiveness, as these bonds display absorption bands highly sensitive to physical variations in the sample [85].

For DMCg, the 1st derivative alone provided the best predictive performance for both mashed and fresh samples, with ${\text{R}}_{\text{pt}}$ = 0.80 and ${\text{RMSE}}_{\text{pt}}$ = 1.20 for mashed samples, and ${\text{R}}_{\text{pt}}$ = 0.73 and ${\text{RMSE}}_{\text{pt}}$ = 1.42 for fresh samples. These results suggest that this pretreatment effectively emphasizes subtle spectral variations associated primarily with water content and volatile compounds, which may undergo rapid loss during root handling, causing minor structural changes not captured without sensitive spectral transformations. The first derivative minimizes baseline shifts and high-frequency noise while highlighting absorption bands characteristic of key chemical constituents [86]. As reported by Medeiros et al. [87] this method can substantially modify NIRS spectral behavior, emphasizing critical regions for calibration. Because near-infrared radiation interacts with molecular vibrations of O–H, C–H, and N–H bonds, variations in water content and structural composition are directly reflected in the corresponding spectral regions. Accordingly, the success of the first derivative in predicting DMCg likely stems from its ability to separate relevant chemical signals from physical or instrumental interferences [22,85,88].

The observed differences in optimal pretreatments between StC and DMCo (SG + SNV) versus DMCg (1st derivative) reinforce the notion that no single spectral pretreatment is universally effective across all traits and datasets. Although spectral pretreatments can enhance relevant differences between samples and improve signal quality [89,90] an inappropriate choice may inadvertently remove useful chemometric information or introduce noise by disrupting critical spectral patterns, compromising both interpretability and model robustness [35]. Consequently, pretreatment selection should not be arbitrary or standardized. It must carefully consider the nature of the target trait, the calibration method, and the objectives of the analysis. Effective strategies amplify relevant spectral signals without introducing artificial noise, maximizing predictive performance while preserving spectral integrity. Building robust, reliable models therefore requires pretreatment approaches that are evidence-based, systematically evaluated, and tailored to the characteristics of each dataset.

### The role of sample preparation in optimizing NIRS predictions

Sample type was consistently found to exert a direct influence on model accuracy. Regardless of the calibration algorithm used (PLS, SVM, or XGB), mashed samples consistently provided higher predictive performance than fresh samples across both calibration and external validation stages (Figs 3 and 4). During calibration, models constructed with mashed samples exhibited high $R_{C}$ and low ${RMSE}_{C}$, particularly for DMCo. For example, the PLS model for mashed samples achieved $R_{C}$ = 0.97 and ${RMSE}_{C}$ = 0.91, indicating near-perfect fit and minimal error, compared to $R_{C}$ = 0.75 and ${RMSE}_{C}$ = 2.15 for fresh samples. The SVM and XGB models also produced high $R_{C}$ values for DMCo (≥0.96), though their ${RMSE}_{C}$ values were slightly higher (0.96 and 1.02, respectively) than those of PLS (Fig 2). In contrast, models calibrated with fresh samples exhibited lower performance, with $R_{C}$ ranging from 0.74 (SVM) to 0.80 (XGB) and ${RMSE}_{C}$ between 1.93 and 2.19. Calibration performance for DMCg and StC was slightly lower than for DMCo, with $R_{C}$ values between 0.78 and 0.84, with PLS slightly outperforming the other algorithms. Nonetheless, these values still exceeded those observed for fresh samples, which ranged from 0.57 to 0.75. Similar results have been reported in the literature. Ikeogu et al. [31] observed significantly higher calibration performance using mashed root samples for dry matter content with a Vis/NIRS spectrometer ($R_{C}$: 0.96–0.99 for mashed vs. 0.86–0.96 for fresh samples). Hershberger et al. [18] also found that ground root samples out-performed sliced samples in SCiO NIRS spectroscopy, with $R_{C}$ values ranging from 0.63 to 0.89 for ground samples versus 0.62 to 0.79 for sliced roots. These findings confirm that preparation methods that enhance intra-sample homogeneity, such as grinding, yield more representative spectra and more stable predictive models.

In external validation, models trained on mashed samples consistently maintained superior predictive performance relative to fresh samples, independent of the calibration algorithm used (Fig 3). For DMCo, the SVM model with mashed samples achieved $R_{v}$ = 0.95 and ${RMSE}_{V}$ = 1.66, compared to $R_{v}$ = 0.78 and ${RMSE}_{V}$ = 2.14 for fresh samples. The PLS and XGB models also performed robustly, although with slightly higher ${RMSE}_{V}$ values. These results highlight the stability of spectra obtained from homogeneous samples, particularly for DMCo, likely due to reduced interference from surface moisture variability. For DMCg and StC, although $R_{v}$ values were lower than for DMCo, models calibrated with mashed samples consistently outperformed those using fresh samples. For example, $R_{v}$ for StC ranged from 0.81 (SVM and XGB) to 0.82 (PLS), versus 0.56–0.60 for fresh samples. This trend was also reflected in ${RPIQ}_{V}$ and ${RPD}_{V}$, which were higher for mashed samples, reaching 3.74 and 2.81, respectively, indicating practical applicability of models calibrated with mashed roots. Despite the overall superior performance of mashed samples, a slightly higher ${bias}_{V}$ was observed, suggesting a tendency to overestimate predicted values. This bias may result from texture standardization and reduced surface heterogeneity, which, while improving overall accuracy, can introduce systematic shifts in regression. Nevertheless, high $R_{V}^{2}$, ${RPIQ}_{V}$, and ${RPD}_{V}$ values indicate that models calibrated with mashed samples can be effectively integrated into breeding workflows. Future studies should explore adjustments as additional data become available to reduce bias and expand application under field conditions.

Root maceration promotes removal of heterogeneous compound distributions within plant tissue, producing more uniform samples in physicochemical terms [31,41]. This homogenization enhances spectral measurement consistency, reduces noise, and improves the extraction of relevant spectral signals. The consistently superior performance of models calibrated with mashed samples across all traits suggests that this preparation method should be preferred in NIRS-assisted phenotyping strategies. Conversely, fresh samples, with their complex gradients in moisture, texture, and composition, can introduce variability that compromises model accuracy, particularly when using portable spectrometers with limited spectral ranges. Nevertheless, results from fresh samples remain valuable. Among the algorithms tested, XGB showed relatively superior performance with fresh samples, representing a promising alternative when sample processing is impractical. These findings are particularly relevant for breeding programs, demonstrating that, even under common field constraints, reasonable prediction accuracy can be achieved by selecting robust algorithms. However, sample type remains a major determinant of NIRS prediction accuracy. While fresh samples offer logistical advantages for rapid, non-destructive analyses, their impact on spectral quality and model performance must be carefully considered, especially in critical applications such as selecting superior genotypes, ensuring product traceability, or implementing quality control in agro-industrial supply chains.

### Comparative performance of calibration models using fresh and mashed root samples

The use of machine learning models to NIRS spectral data has shown strong potential for predicting chemical and physical traits in cassava roots [33,91,92]. Comparative analysis of PLS, SVM, and XGB models revealed substantial variation in predictive performance, influenced by both sample type (mashed or fresh) and the target trait (StC, DMCo, and DMCg). Overall, the PLS model exhibited consistent and superior performance with mashed samples, particularly for DMCo, achieving the highest predictive accuracy ($R_{C}$ = 0.97), coefficient of determination ($R_{CV}^{2}$ = 0.94), and the lowest ${RMSE}_{C}$ (Fig 2). This suggests that the linear PLS approach is highly effective when applied to spectra from homogenized samples, where physical uniformity minimizes interference from factors unrelated to chemical composition. The robustness of PLS in this context can be attributed to its capacity to extract latent variables that explain the majority of variance in spectral data, even under multicollinearity conditions [84,93].

In addition to conventional metrics, high ${RPD}_{C}$ and ${RPIQ}_{C}$ values further confirm the reliability of PLS for mashed samples. For DMCo, ${RPD}_{C}$ exceeded 4.2 and ${RPIQ}_{C}$ surpassed 5.9, indicating excellent model trust-worthiness [94–97]. These indices, which account for actual sample variability relative to prediction error, are particularly informative for assessing model robustness in practical applications. Similar results were reported by Zhao et al. [98] in predicting lignocellulosic content across 148 samples of rice, maize, wheat, soybean, sorghum, rapeseed, and peanut straw, where PLS significantly outperformed SVM ($R_{C}$ = 0.8983; ${RMSE}_{C}$ = 0.6299; ${RPD}_{C}$ = 3.49). Likewise, Nakawajana and Posom [99] used an FT-NIRS spectrometer (MPA, Bruker, Germany) to predict energy-related traits in ground cassava rhizomes, finding that both PLS and SVM achieved high accuracy, with PLS slightly superior ($R_{C}$ = 0.90–0.98 vs. 0.84–0.90). Differences in performance were attributed to experimental conditions, including clone number, environment, sample type, and genetic variability, emphasizing the influence of context on model outcomes. Conversely, fresh samples, characterized by greater physical heterogeneity due to natural gradients in DMC and StC along the root (from base to tip and from core to peel), were best modeled by XGB, which consistently outperformed other algorithms across all traits (Fig 2). This superiority was evident not only in $R_{C}$ and ${RMSE}_{C}$ but also in ${RPD}_{C}$ and ${RPIQ}_{C}$, highlighting the efficacy of non-linear algorithms in identifying latent patterns in complex spectral data. The XGB’s ability to model intricate interactions and subtle spectral variations [61] makes it particularly suitable for scenarios where linear methods such as PLS lose accuracy due to high intrinsic variability. Sousa et al. [23], examining 1,127 F_2_ seeds from 77 controlled F_1_ crosses, demonstrated the influence of NIRS device type on model performance. Using the NIRFlex N-500, $R_{C}$ ranged from 0.86 (RF) to 0.92 (XGB), reflecting XGB’s ability to capture complex patterns in high-resolution spectra. In contrast, using the lower-resolution SCiO device, $R_{C}$ ranged from 0.87 (C5.0 decision tree) to 0.89 (RF and XGB). These findings indicate that while spectral quality influences model performance, XGB can maintain robust accuracy even under challenging instrumental conditions, supporting its applicability in cost-effective portable devices. This capacity enhances its potential for large-scale, rapid, non-destructive field applications in breeding programs.

The SVM model showed intermediate performance between PLS and XGB, with slight advantage over XGB only in mashed samples for DMCg and DMCo. However, its predictive accuracy declined markedly for fresh samples, consistently underperforming across all traits, as reflected in lower ${RPD}_{C}$ (1.19–1.68) and ${RPIQ}_{C}$ (1.75–2.29). This limitation may stem from the standard radial basis function (RBF) kernel configuration (C = 1) without targeted hyperparameter tuning. The lack of fine-tuning likely constrained the model’s flexibility in handling the non-systematic noise typical of structurally and compositionally heterogeneous fresh samples, as evidenced by the lowest $R_{C}$ observed (0.57) for StC prediction. Although non-linear models like SVM often excel in predicting complex traits, their performance can vary considerably depending on sample type and target variable. Gürbüz et al. [100] reported that SVM outperformed PLS for oil content prediction in 50 maize seed samples ($R_{C}$ = 0.95 vs. 0.86; ${RMSE}_{C}$ = 0.46 vs. 0.76), whereas Mbanjo et al. [35] found PLS and SVM to perform comparably in predicting StC for 344 African cassava clones using SCiO spectra from fresh roots ($R_{C}$≈ 0.89; ${RMSE}_{C}$≈ 2.07). These observations underscore that algorithm selection should consider the biological matrix and target trait rather than assuming a universally superior model.

Overall, these results demonstrate that no single model is universally optimal. Algorithm selection depends on spectral quality, sample structure, and the characteristics of the target trait. Linear models such as PLS are highly effective when the spectrum–composition relationship is direct and robust, as in mashed samples. More complex algorithms, such as XGB, excel when flexibility is required to capture non-linear patterns, as in fresh samples. These findings emphasize that successful calibration strategies must account for sample type from the outset and suggest the potential value of hybrid or adaptive approaches, combining different algorithms based on spectral context. By leveraging the strengths and limitations of each model, NIR spectroscopy can be optimally applied to predict cassava root quality traits, even in the presence of inherent variability under real-world sampling conditions.

### External validation highlights the robustness of PLS in spectral prediction

External validation revealed that the predictive performance of the evaluated models (PLS, SVM, and XGB) varied depending on the phenotypic trait. Nonetheless, regardless of the target trait or sample type, PLS consistently demonstrated superior performance, highlighting its robustness and stability across all tested scenarios (Fig 3). High $R_{V}$ and $R_{V}^{2}$ values, frequently accompanied by ${RPD}_{V}$ and ${RPIQ}_{V}$ exceeding thresholds indicative of good or excellent predictive ability [22,95], underscore PLS’s capacity to generalize from spectral data. The prediction of StC in mashed samples illustrates this consistency. All three models produced similar $R_{V}$ values, ranging from 0.81 (SVM and XGB) to 0.82 (PLS), with $R_{V}^{2}$ values between 0.66 (SVM and XGB) and 0.67 (PLS) (Fig 3). The ${RMSE}_{V}$ values were identical across models (1.99), reinforcing the overall consistency in estimating this parameter. Slight differences were observed in ${RPIQ}_{V}$, ranging from 1.90 (SVM) to 2.01 (PLS), suggesting a modest advantage for PLS in terms of lower error and greater stability. A similar pattern was observed for DMCg, where PLS exhibited the highest $R_{V}$, $R_{V}^{2}$, and ${RPIQ}_{V}$ values and the lowest ${RMSE}_{V}$ ($R_{V}$ = 0.64, $R_{V}^{2}$ = 0.41, ${RMSE}_{V}$ = 1.66, ${RPIQ}_{V}$ = 1.88) relative to SVM and XGB. For DMCo, $R_{V}$ values were comparable between PLS and SVM (0.95); however, SVM showed slightly higher predictive accuracy, with lower ${RMSE}_{V}$ (1.66) and higher $R_{V}^{2}$, ${RPD}_{V}$, and ${RPIQ}_{V}$ values (0.91, 2.81, and 3.74, respectively).

For fresh samples, similar trait-dependent patterns emerged. The PLS outperformed the other models in predicting StC and DMCg (Fig 3), showing particular efficiency for DMCg ($R_{V}$ = 0.47, ${RMSE}_{V}$ = 1.93, $R_{V}^{2}$ = 0.22). For StC, SVM achieved a slightly higher $R_{V}$ (0.60) than PLS (0.58); however, PLS demonstrated lower ${RMSE}_{V}$ and higher ${RPIQ}_{V}$ (1.98 and 1.66, respectively), indicating superior predictive ability. Likewise, for DMCo prediction using fresh samples, SVM exhibited slightly higher accuracy than PLS and XGB, with higher $R_{V}$, ${RPD}_{V}$, and ${RPIQ}_{V}$ (0.78, 1.58, and 2.18) and lower ${RMSE}_{V}$ (2.14). Previous studies support these findings. Freitas et al. [92] evaluated 238 cassava accessions for resistance to frog skin disease using NIR spectroscopy (NIRFlex N-500), reporting high predictive accuracy for SVM and BGLM (0.99 and 0.98, respectively), surpassing PLS (0.91). These results highlight the advantages of non-linear algorithms for complex classification tasks, even in rapid spectral screening. Conversely, Sánchez et al. [32] demonstrated that traditional approaches, such as PLS, can achieve excellent performance in predicting quantitative traits. Evaluating 3,418 NIR spectra collected over four years with a FOSS 6500 spectrometer, they reported high predictive ability for DMCo in fresh cassava samples, with a standard error of prediction (SEP) of 1.42 and a coefficient of determination of 0.946. Bantadjan et al. [3] explored two portable spectrometers, STS-NIR (Ocean Optics, 650–1100 nm) and Maya2000 Pro-NIR (Ocean Optics, 720–1050 nm), for StC prediction in 200 fresh cassava roots. Using PLS, prediction accuracy varied substantially depending on spectral pretreatment and wavelength range ($R_{C}$ = 0.689–0.918 at 720–1050 nm; 0.773–0.928 at 850–1150 nm), demonstrating the robustness of PLS even with limited spectral coverage.

Overall, these results indicate that all three models can generate effective predictive models using NIR spectra, particularly for mashed samples, although performance varies depending on the target trait. The PLS model proved especially effective for StC and DMCg, confirming its versatility for spectral phenotyping programs. The SVM model emerged as a competitive alternative for DMCo prediction, demonstrating that machine learning approaches can provide targeted advantages. The balance of predictive accuracy and stability exhibited by PLS positions it as a reliable choice for applications requiring robust generalization.

### Consistency of spectral models in classifying cassava clones

Selection of superior clones in breeding programs requires consistent and reliable evaluation of key traits [101]. Traditionally, these assessments rely on methods that are labor-intensive, time-consuming, and costly, such as semi-industrial starch quantification or dry matter determination via oven-drying. While highly accurate, these approaches demand substantial energy input to dry samples to constant weight, resulting in low daily throughput, which makes them impractical for high-throughput phenotyping during early selection stages [18,19]. Moreover, many traditional phenotyping methods are destructive, posing limitations when working with scarce or genetically valuable materials. In this context, rapid and non-destructive techniques, such as near-infrared spectroscopy (NIRS), have emerged as strategic tools for high-throughput screening at reduced cost. Advances in optical components, computational power, and robust chemometric approaches now allow complex information to be efficiently and accurately extracted from spectral data [18,24,31,101–103].

Beyond quantitative trait prediction, NIRS can be applied for genotype classification using agreement metrics such as the kappa index. This index quantifies the concordance between predicted and observed classifications, correcting for chance agreement, and provides a practical and objective measure to evaluate the consistency of selection decisions based on spectral models [94,104]. It thereby enhances confidence in identifying superior clones and accelerates genetic progress while reducing costs. The models evaluated (PLS, SVM, and XGB) exhibited differences in their efficiency to classify the top 20% of clones, as assessed by the kappa index (Fig 4). Kappa values range from 0, indicating agreement equivalent to chance, to 1, indicating perfect agreement [94,105]. Despite variations among models, all achieved high kappa values, reflecting both their accuracy and the low likelihood of random classification of superior clones. This underscores the robustness of the models and the reliability of NIRS-based phenotypic selection.

For starch content (StC), predicted and observed values revealed high agreement in identifying the top 20% of clones for both mashed and fresh samples (Fig 4). In mashed samples, XGB performed best (kappa = 0.99), followed by PLS (0.96) and SVM (0.91), indicating that all models correctly classified the majority of samples. Pairwise comparisons also showed strong concordance among models (PLS–SVM: 0.91; PLS–XGB: 0.97; SVM–XGB: 0.92), suggesting that models based on distinct mathematical principles produce highly consistent predictions. In fresh samples, kappa values ranged from 0.81 to 1.0, indicating near-perfect agreement. The PLS model achieved kappa = 1.0, reflecting perfect alignment between predictions and observations, while SVM and XGB scored slightly lower (kappa = 0.99) (Fig 4A). Pairwise model comparisons yielded kappa = 1.0 for all combinations, highlighting the convergent reliability of different algorithms. For dry matter content (DMCg), especially in mashed samples, the models exhibited remarkable consistency, with kappa values frequently approaching or reaching 1 (Fig 4C). Even in fresh samples, where physical heterogeneity is higher, models maintained high performance (kappa ≈ 1), demonstrating that structural variability did not impair the algorithms’ ability to consistently identify top-performing clones. Despite these high kappa values, DMCg showed lower $R_{V}$ values compared to other traits (0.2–0.41). Peternelli et al. [106] highlighted the utility of kappa as a complementary metric to accuracy, noting that well-fitted models can yield high kappa values even when the coefficient of determination is moderate.

For oven-dry matter content (DMCo), predictive models also showed high concordance for both mashed and fresh samples (Fig 4B). In mashed samples, SVM and XGB achieved perfect agreement (kappa = 1.0), while PLS was slightly lower (kappa = 0.79). Nonetheless, pairwise comparisons among models (PLS–SVM, PLS–XGB, SVM–XGB) resulted in kappa = 1.0, indicating complete alignment across algorithms. In fresh samples, kappa values remained high, with SVM highest (0.95), followed by PLS (0.88) and XGB (0.89), all within the range considered perfect agreement [106]. Peternelli et al. [106] reported similar trends using PLS and FT-NIR spectra in sugarcane, highlighting that predicting traits with higher structural variability (e.g., fiber content) is more challenging, and providing a benchmark for assessing model robustness. Overall, even when selecting only the top 20% of clones, the observed kappa values demonstrate the efficiency of the algorithms in classifying superior genotypes. These results confirm NIRS as an efficient, non-destructive tool for large-scale phenotyping, enabling rapid, cost-effective, and reproducible evaluation of extensive sample sets. The NIRS facilitates early identification of promising genotypes, reduces reliance on laborious laboratory methods, and provides a reliable foundation for informed decision-making in breeding programs.

### Prospects of NIRS for cassava breeding

The NIR spectroscopy has emerged as a strategic tool to overcome the bottlenecks in phenotyping complex traits within cassava breeding programs [18,31,91]. Historically, phenotypic evaluation of traits such as dry matter content (DMC) and starch content (StC) has been limited to the later stages of selection due to reliance on labor-intensive, costly, and low-throughput laboratory methods, such as oven-drying samples to constant weight [15,18,19]. To circumvent these constraints, simpler and faster alternatives, such as gravimetric estimations based on empirical equations, have been applied in both research and commercial settings. While practical, these approaches carry a hidden cost: the premature exclusion of promising genotypes that cannot be accurately evaluated during the early stages of selection.

In this context, NIRS has become a powerful ally in cassava breeding. This technology enables rapid, non-destructive, and high-throughput analysis of large populations, facilitating efficient phenotyping [32,41,107]. Its key advantage lies in the capacity to perform measurements directly in the field without prior laboratory processing while simultaneously predicting multiple traits with accuracy comparable to conventional laboratory methods, provided that properly calibrated spectrometers and predictive models are employed [35]. Recent studies illustrate this potential, such as Chaiareekitwat et al. [36] who reported prediction accuracies of up to 0.98 for DMC, whereas Maraphum et al. [66] observed values up to 0.68. Similarly, Rosales et al. [108] reported prediction accuracies near 0.75 for total carotenoid content (TCC). These findings underscore the effectiveness of NIRS in transforming phenotypic selection, making it faster, more accessible, and compatible with the scale and demands of modern breeding programs.

The ability to assess hundreds of samples during early selection stages, impractical with conventional methods, represents a paradigm shift in cassava breeding. The NIR spectroscopy allows for the evaluation of a substantially larger number of genotypes directly in the field, preserving sample integrity and minimizing risks associated with transport and storage [18,109]. By enabling earlier selection decisions, NIRS reduces dependence on laboratory infrastructure and associated costs, thereby allowing for expanded evaluation across multiple sites. Adoption of this technology can significantly shorten phenotypic assessment times, freeing human and operational resources for other breeding activities. Its efficiency is reflected not only in reduced time and costs but also in the ability to make earlier selection decisions based on reliable predicted values. Consistency between predicted and observed values has demonstrated satisfactory performance of the developed models [92,106].

Despite these advances, challenges remain for the large-scale adoption of NIRS. Instrument variability, the influence of sample type (fresh versus processed) [18,31,32], and the need for model validation across diverse populations and environmental conditions highlight areas requiring further attention. In Brazil, for instance, studies employing locally calibrated models remain scarce, limiting the direct application of models developed elsewhere.

Nevertheless, considering the portability and connectivity of the SCiO spectrometer, its use under field conditions is entirely feasible with minor operational adjustments. In this study, roots were washed and peeled prior to spectral acquisition, a procedure widely adopted in previous NIRS studies on cassava [31,35,38]. Such preparation is essential to ensure high reproducibility and calibration accuracy, as processed samples (washed, peeled, or mashed) typically yield more robust predictive models than intact fresh roots. Hershberger et al. [18] reported that spectra collected from cut but unpeeled roots can still produce predictive models with substantial potential; however, prediction accuracy was higher for peeled and mashed samples. For future applications, the sensor could potentially be applied directly to freshly harvested roots, provided that excess soil is superficially removed. The manufacturer offers a plastic “light shield” that can be attached to the spectrometer to minimize ambient light interference. Additionally, spectral acquisition could be conducted under shade using a protective cover or simple canopy, which may further reduce the impact of external light, a hypothesis that warrants experimental verification. It should be noted, however, that deploying the SCiO in remote locations may face logistical limitations, as the device requires an active license and internet connection for operation [35]. Nevertheless, the combination of portability, rapid measurement, and integration with mobile devices positions NIRS as a promising tool for non-destructive screening in cassava breeding programs. This approach has the potential to reduce costs, time, and labor while enabling broader and faster evaluations across multiple sites. Moreover, the development of new portable devices that do not require an internet connection is likely to further expand the practical applicability of NIRS in remote breeding programs.

## Conclusions

This study demonstrated the potential of portable NIRS devices (SCiO) for non-destructive prediction of quality traits in cassava roots using both mashed and fresh samples. Calibration of predictive models with different algorithms and spectral preprocessing methods revealed that mashed samples generally provide superior predictive performance, particularly when combined with SG + SNV pre-treatment for StC and DMCo, and first-derivative transformation for DMCg. Among the models tested, PLS consistently achieved high correlation coefficients and low RMSE values, distinguishing itself as the most effective.

These findings have important implications for cassava breeding programs, highlighting the feasibility of adopting rapid, cost-effective, and non-destructive approaches for large-scale phenotypic screening. High concordance between predicted and observed selections further validates the reliability of this approach, even in the presence of the complex chemical and physical characteristics of cassava roots. Consequently, portable NIRS sensors can significantly accelerate the selection of superior genotypes, making them applicable from the earliest stages of breeding. Future research may explore integration of these models into digital field platforms and their adaptation to other crops facing similar phenotyping challenges.

## References

[1] Ceballos H, Iglesias CA, Pérez JC, Dixon AGO. Cassava breeding: opportunities and challenges. Plant Mol Biol. 2004;56(4):503–16. doi: 10.1007/s11103-004-5010-5 15630615

[2] Borku AW. Cassava (*Manihot esculenta* Crantz): its nutritional composition insights for future research and development in Ethiopia. Discov Sustain. 2025;6(1). doi: 10.1007/s43621-025-00996-2

[3] Bantadjan Y, Rittiron R, Malithong K, Narongwongwattana S. Establishment of an Accurate Starch Content Analysis System for Fresh Cassava Roots Using Short-Wavelength Near Infrared Spectroscopy. ACS Omega. 2020;5(25):15468–75. doi: 10.1021/acsomega.0c01598 32637821 PMC7331049

[4] Wang Z, Mhaske P, Farahnaky A, Kasapis S, Majzoobi M. Cassava starch: Chemical modification and its impact on functional properties and digestibility, a review. Food Hydrocolloids. 2022;129:107542. doi: 10.1016/j.foodhyd.2022.107542 33966555

[5] Odedeyi T, Rabbi I, Poole C, Darwazeh I. Estimation of starch content in cassava based on coefficient of reflection measurement. Front Food Sci Technol. 2022;2. doi: 10.3389/frfst.2022.878023

[6] Charles A, Sriroth K, Huang T. Proximate composition, mineral contents, hydrogen cyanide and phytic acid of 5 cassava genotypes. Food Chemistry. 2005;92(4):615–20. doi: 10.1016/j.foodchem.2004.08.024

[7] Gomes E, Souza SR de, Grandi RP, Silva R da. Production of thermostable glucoamylase by newly isolated Aspergillus flavus A 1.1 and Thermomyces lanuginosus A 13.37. Braz J Microbiol. 2005;36(1). doi: 10.1590/s1517-83822005000100015

[8] Ngiki YU, Igwebuike JU, Moruppa SM. Utilisation of cassava products for poultry feeding: a review. Int J Sci Technol. 2014;2:48–59.

[9] Tonukari NJ. Cassava and the future of starch. Electron J Biotechnol. 2004;7(1). doi: 10.2225/vol7-issue1-fulltext-9

[10] Butarelo SS, Beleia A, Fonseca IC de B, Ito KC. Hidratação de tecidos de raízes de mandioca (*Manihot esculenta* Crantz.) e gelatinização do amido durante a cocção. Ciênc Tecnol Aliment. 2004;24(3):311–5. doi: 10.1590/s0101-20612004000300001

[11] Moorthy S, Padmaja GA. A rapid titrimetric method for the determination of starch content of cassava tubers. J Root Crops. 2002;28:30–7.

[12] Santos VDS, Santos MLMD, Sasaki FFC, Oliveira LAD, Ledo CADS. Agronomic Characteristics, Cooking And Postharvest Conservation For Selecting Sweet Cassava Clones. Rev Caatinga. 2022;35(3):730–8. doi: 10.1590/1983-21252022v35n324rc

[13] Kawano K, Fukuda WMG, Cenpukdee U. Genetic and Environmental Effects on Dry Matter Content of Cassava Root1. Crop Science. 1987;27(1):69–74. doi: 10.2135/cropsci1987.0011183x002700010018x

[14] Silva RM da, Fernandes AM, Leonel M, Pelvine RA, Figueiredo RT de, Rangel MAS, et al. Measurement of Dry Matter and Starch in Modern Cassava Genotypes during Long Harvest Cycles. Horticulturae. 2023;9(7):733. doi: 10.3390/horticulturae9070733

[15] Teye E, Asare AP, Amoah RS, Tetteh JP. Determination of the dry matter content of cassava (*Manihot esculenta*, Crantz) tubers using specific gravity method. J Agric Biol Sci. 2011;6:23–8.

[16] Pola AC, Moreto AL, Nunes E da C. Matéria seca em raízes de mandioca determinada pelos métodos da balança hidrostática e de secagem em estufa. RAC. 2020;33(1):56–60. doi: 10.52945/rac.v33i1.533

[17] Vasconcelos LM, Brito AC, Carmo CD, Oliveira PHGA, Oliveira EJ. Phenotypic diversity of starch granules in cassava germplasm. Genet Mol Res. 2017;16(2):10.4238/gmr16029276. doi: 10.4238/gmr16029276 28407178

[18] Hershberger J, Mbanjo EGN, Peteti P, Ikpan A, Ogunpaimo K, Nafiu K, et al. Low‐cost, handheld near‐infrared spectroscopy for root dry matter content prediction in cassava. The Plant Phenome Journal. 2022;5(1). doi: 10.1002/ppj2.20040

[19] Teles F. An easy technique for rapid determination of dry-matter content in cassava roots (*Manihot esculenta* Crantz). Food Chemistry. 1993;47(4):375–7. doi: 10.1016/0308-8146(93)90180-n

[20] Cobb JN, Declerck G, Greenberg A, Clark R, McCouch S. Next-generation phenotyping: requirements and strategies for enhancing our understanding of genotype-phenotype relationships and its relevance to crop improvement. Theor Appl Genet. 2013;126(4):867–87. doi: 10.1007/s00122-013-2066-0 23471459 PMC3607725

[21] Reynolds M, Chapman S, Crespo-Herrera L, Molero G, Mondal S, Pequeno DNL, et al. Breeder friendly phenotyping. Plant Sci. 2020;295:110396. doi: 10.1016/j.plantsci.2019.110396 32534615

[22] Williams P, Norris K. Near-infrared technology in the agricultural and food industries. 2nd ed. St. Paul, MN: American Association of Cereal Chemists; 2001.

[23] Sousa MBE, Filho JSS, de Andrade LRB, de Oliveira EJ. Near-infrared spectroscopy for early selection of waxy cassava clones via seed analysis. Front Plant Sci. 2023;14:1089759. doi: 10.3389/fpls.2023.1089759 36755702 PMC9900181

[24] Lu G, Huang H, Zhang D. Application of near-infrared spectroscopy to predict sweetpotato starch thermal properties and noodle quality. J Zhejiang Univ Sci B. 2006;7(6):475–81. doi: 10.1631/jzus.2006.B0475 16691642 PMC1474002

[25] Stuart BH. Infrared Spectroscopy: Fundamentals and Applications. Analytical Techniques in the Sciences. Wiley; 2004. doi: 10.1002/0470011149

[26] Basati Z, Jamshidi B, Rasekh M, Abbaspour-Gilandeh Y. Detection of sunn pest-damaged wheat samples using visible/near-infrared spectroscopy based on pattern recognition. Spectrochim Acta A Mol Biomol Spectrosc. 2018;203:308–14. doi: 10.1016/j.saa.2018.05.123 29879646

[27] Ssali Nantongo J, Serunkuma E, Burgos G, Nakitto M, Davrieux F, Ssali R. Machine learning methods in near infrared spectroscopy for predicting sensory traits in sweetpotatoes. Spectrochim Acta A Mol Biomol Spectrosc. 2024;318:124406. doi: 10.1016/j.saa.2024.124406 38759574

[28] López A, Arazuri S, García I, Mangado J, Jarén C. A review of the application of near-infrared spectroscopy for the analysis of potatoes. J Agric Food Chem. 2013;61(23):5413–24. doi: 10.1021/jf401292j 23647358

[29] Esteve Agelet L, Hurburgh CR Jr. Limitations and current applications of Near Infrared Spectroscopy for single seed analysis. Talanta. 2014;121:288–99. doi: 10.1016/j.talanta.2013.12.038 24607140

[30] Alander JT, Bochko V, Martinkauppi B, Saranwong S, Mantere T. A Review of Optical Nondestructive Visual and Near-Infrared Methods for Food Quality and Safety. International Journal of Spectroscopy. 2013;2013:1–36. doi: 10.1155/2013/341402

[31] Ikeogu UN, Davrieux F, Dufour D, Ceballos H, Egesi CN, Jannink J-L. Rapid analyses of dry matter content and carotenoids in fresh cassava roots using a portable visible and near infrared spectrometer (Vis/NIRS). PLoS One. 2017;12(12):e0188918. doi: 10.1371/journal.pone.0188918 29228026 PMC5724885

[32] Sánchez T, Ceballos H, Dufour D, Ortiz D, Morante N, Calle F, et al. Prediction of carotenoids, cyanide and dry matter contents in fresh cassava root using NIRS and Hunter color techniques. Food Chem. 2014;151:444–51. doi: 10.1016/j.foodchem.2013.11.081 24423555

[33] Abubakar M, Wasswa P, Masumba E, Ongom P, Mkamilo G, Kanju E, et al. Use of low cost near-infrared spectroscopy, to predict pasting properties of high quality cassava flour. Sci Rep. 2024;14(1):17130. doi: 10.1038/s41598-024-67299-w 39054362 PMC11272776

[34] Zhang Y, Nie L, Sun J, Hong Y, Yan H, Li M, et al. Impacts of Environmental Factors on Pasting Properties of Cassava Flour Mediated by Its Macronutrients. Front Nutr. 2020;7:598960. doi: 10.3389/fnut.2020.598960 33324670 PMC7725801

[35] Nkouaya Mbanjo EG, Hershberger J, Peteti P, Agbona A, Ikpan A, Ogunpaimo K, et al. Predicting starch content in cassava fresh roots using near-infrared spectroscopy. Front Plant Sci. 2022;13:990250. doi: 10.3389/fpls.2022.990250 36426140 PMC9679500

[36] Chaiareekitwat S, Mahayothee B, Rungpichayapichet P, Khuwijitjaru P, Nagle M, Latif S, et al. The potential of near–infrared spectroscopy as a rapid method for quality evaluation of cassava leaves and roots. Journal of Food Composition and Analysis. 2024;126:105913. doi: 10.1016/j.jfca.2023.105913

[37] Namakula BF, Nuwamanya E, Kanaabi M, Wembambazi E, Kawuki RS. Predicting starch content of cassava with near infrared spectroscopy in Ugandan cassava germplasm. Journal of Near Infrared Spectroscopy. 2023;31(5):256–62. doi: 10.1177/09670335231194739

[38] Bandeira E Sousa M, Morales CFG, Mbanjo EGN, Egesi C, de Oliveira EJ. Near infrared spectroscopy for cooking time classification of cassava genotypes. Front Plant Sci. 2024;15:1411772. doi: 10.3389/fpls.2024.1411772 39070913 PMC11272462

[39] dos Santos CAT, Lopo M, Páscoa RNMJ, Lopes JA. A review on the applications of portable near-infrared spectrometers in the agro-food industry. Appl Spectrosc. 2013;67(11):1215–33. doi: 10.1366/13-07228 24160873

[40] Ptushenko VV, Linev VN. A Review of the Dawn of Benchtop EPR Spectrometers-Innovation That Shaped the Future of This Technology. Molecules. 2022;27(18):5996. doi: 10.3390/molecules27185996 36144728 PMC9503086

[41] Nuwamanya E, Wembabazi E, Kanaabi M, Namakula FB, Katungisa A, Lyatumi I, et al. Development and validation of near-infrared spectroscopy procedures for prediction of cassava root dry matter and amylose contents in Ugandan cassava germplasm. J Sci Food Agric. 2024;104(8):4793–800. doi: 10.1002/jsfa.12966 37665950

[42] Campbell MR, Mannis SR, Port HA, Zimmerman AM, Glover DV. Prediction of Starch Amylose Content Versus Total Grain Amylose Content in Corn by Near‐Infrared Transmittance Spectroscopy. Cereal Chem. 1999;76(4):552–7. doi: 10.1094/cchem.1999.76.4.552

[43] Beć KB, Grabska J, Huck CW. Miniaturized NIR Spectroscopy in Food Analysis and Quality Control: Promises, Challenges, and Perspectives. Foods. 2022;11(10):1465. doi: 10.3390/foods11101465 35627034 PMC9140213

[44] Brereton RG. Introduction to multivariate calibration in analytical chemistry. Analyst. 2000;125(11):2125–54. doi: 10.1039/b003805i

[45] Mishra AR, Karimi D, Ehsani R, Lee WS. Identification of Citrus Greening (HLB) Using a VIS-NIR Spectroscopy Technique. Transactions of the ASABE. 2012;55(2):711–20. doi: 10.13031/2013.41369

[46] Köppen W, Geiger R. Das geographische System der Klimate. Handbuch der Klimatologie. Berlin: Verlag Gebrüder Bornträger; 1936. p. 1–44.

[47] Souza LS, Farias ARN, Mattos PLP, Fukuda WMG. Aspectos socioeconômicos e agronômicos da mandioca. Cruz das Almas: Embrapa Mandioca e Fruticultura Tropical; 2006.

[48] de Carvalho RRB, Bandeira E Sousa M, de Oliveira LA, de Oliveira EJ. Phenotypic diversity and selection in biofortified cassava germplasm for yield and quality root traits. Euphytica. 2022;218(12):173. doi: 10.1007/s10681-022-03125-6 36405300 PMC9668781

[49] Sánchez T, Salcedo E, Ceballos H, Dufour D, Mafla G, Morante N, et al. Screening of Starch Quality Traits in Cassava (*Manihot esculenta* Crantz). Starch Stärke. 2009;61(1):12–9. doi: 10.1002/star.200800058

[50] Bian X, Wang K, Tan E, Diwu P, Zhang F, Guo Y. A selective ensemble preprocessing strategy for near-infrared spectral quantitative analysis of complex samples. Chemometrics and Intelligent Laboratory Systems. 2020;197:103916. doi: 10.1016/j.chemolab.2019.103916

[51] Gerretzen J, Szymańska E, Jansen JJ, Bart J, van Manen H-J, van den Heuvel ER, et al. Simple and Effective Way for Data Preprocessing Selection Based on Design of Experiments. Anal Chem. 2015;87(24):12096–103. doi: 10.1021/acs.analchem.5b02832 26632985

[52] Fearn T, Riccioli C, Garrido-Varo A, Guerrero-Ginel JE. On the geometry of SNV and MSC. Chemometrics and Intelligent Laboratory Systems. 2009;96(1):22–6. doi: 10.1016/j.chemolab.2008.11.006

[53] Li P, Zhang X, Li S, Du G, Jiang L, Liu X, et al. A Rapid and Nondestructive Approach for the Classification of Different-Age Citri Reticulatae Pericarpium Using Portable Near Infrared Spectroscopy. Sensors (Basel). 2020;20(6):1586. doi: 10.3390/s20061586 32178312 PMC7146621

[54] Savitzky A, Golay MJE. Smoothing and Differentiation of Data by Simplified Least Squares Procedures. Anal Chem. 1964;36(8):1627–39. doi: 10.1021/ac60214a047

[55] Stevens A, Ramirez-Lopez L. An introduction to the prospectr package. 2013.

[56] Tukey JW. Exploratory data analysis. Massachusetts, USA: Addison-Wesley; 1977.

[57] Abdi H. Partial least squares regression and projection on latent structure regression (PLS Regression). WIREs Computational Stats. 2010;2(1):97–106. doi: 10.1002/wics.51

[58] Helland I. Partial least squares regression. In: Balakrishnan N, Colton T, Everitt B, Piergorsh W, Ruggeri F, Teugels JL, editors. Wiley StatsRef: Statistics Reference Online. John Wiley & Sons, Ltd; 2014. doi: 10.1002/9781118445112.stat03287

[59] Murukesan MM, Selvaraj A, Balasubramaniam K, Baluprithviraj KN. Evaluation and categorization of raw cassava log quality for structural applications. Matéria (Rio J). 2024;29(3). doi: 10.1590/1517-7076-rmat-2024-0347

[60] Drucker H, Burges CJC, Kaufman L, Smola A, Vapnik V. Support vector regression machines. In: Mozer MC, Jordan MI, Petsche T, editors. Advances in Neural Information Processing Systems. San Mateo, CA: Morgan Kaufmann; 1997. p. 155–61.

[61] Chen T, Guestrin C. XGBoost: A scalable tree boosting system. Proceedings of the 22nd ACM SIGKDD International Conference on Knowledge Discovery and Data Mining. New York, NY, USA: ACM; 2016. p. 785–794. doi: 10.1145/2939672.2939785

[62] Svetnik V, Liaw A, Tong C, Culberson JC, Sheridan RP, Feuston BP. Random forest: a classification and regression tool for compound classification and QSAR modeling. J Chem Inf Comput Sci. 2003;43(6):1947–58. doi: 10.1021/ci034160g 14632445

[63] Luckner M, Topolski B, Mazurek M. Application of XGBoost Algorithm in Fingerprinting Localisation Task. Lecture Notes in Computer Science. Springer International Publishing; 2017. p. 661–71. doi: 10.1007/978-3-319-59105-6_57

[64] R Core Team. R: A Language and Environment for Statistical Computing. Vienna, Austria: R Foundation for Statistical Computing; 2024.

[65] Kuhn M. Building Predictive Models inRUsing thecaretPackage. J Stat Soft. 2008;28(5). doi: 10.18637/jss.v028.i05

[66] Maraphum K, Saengprachatanarug K, Wongpichet S, Phuphuphud A, Posom J. Achieving robustness across different ages and cultivars for an NIRS-PLSR model of fresh cassava root starch and dry matter content. Computers and Electronics in Agriculture. 2022;196:106872. doi: 10.1016/j.compag.2022.106872

[67] Metzger K, Liebisch F, Herrera JM, Guillaume T, Walder F, Bragazza L. The use of visible and near‐infrared spectroscopy for in‐situ characterization of agricultural soil fertility: A proposition of best practice by comparing scanning positions and spectrometers. Soil Use and Management. 2023;40(1). doi: 10.1111/sum.12952

[68] Moura-Bueno JM, Dalmolin RSD, Horst-Heinen TZ, Grunwald S, ten Caten A. Environmental covariates improve the spectral predictions of organic carbon in subtropical soils in southern Brazil. Geoderma. 2021;393:114981. doi: 10.1016/j.geoderma.2021.114981

[69] Cohen J. A Coefficient of Agreement for Nominal Scales. Educational and Psychological Measurement. 1960;20(1):37–46. doi: 10.1177/001316446002000104

[70] Garrick DJ, Taylor JF, Fernando RL. Deregressing estimated breeding values and weighting information for genomic regression analyses. Genet Sel Evol. 2009;41(1):55. doi: 10.1186/1297-9686-41-55 20043827 PMC2817680

[71] Boer MP. Tensor product P-splines using a sparse mixed model formulation. Statistical Modelling. 2023;23(5–6):465–79. doi: 10.1177/1471082x231178591

[72] McHugh ML. Interrater reliability: the kappa statistic. Biochem Med. 2012:276–82. doi: 10.11613/bm.2012.031 PMC3900052 23092060

[73] Sohaib Ali Shah S, Zeb A, Qureshi WS, Arslan M, Ullah Malik A, Alasmary W, et al. Towards fruit maturity estimation using NIR spectroscopy. Infrared Physics & Technology. 2020;111:103479. doi: 10.1016/j.infrared.2020.103479

[74] Zhang X-W, Chen Z-G, Jiao F. Application of the combination method based on RF and LE in near infrared spectral modeling. Spectrochim Acta A Mol Biomol Spectrosc. 2023;289:122247. doi: 10.1016/j.saa.2022.122247 36549073

[75] Li X, Li C. Pretreatment and Wavelength Selection Method for Near-Infrared Spectra Signal Based on Improved CEEMDAN Energy Entropy and Permutation Entropy. Entropy. 2017;19(7):380. doi: 10.3390/e19070380

[76] Chávez AL, Ceballos H, Rodriguez-Amaya DB, Perez JC, Sanchez T, Calle F, et al. J Root Crops. 2008;34:43–9. https://www.researchgate.net/profile/Nelson-Morante/publication/228426829_Sampling_variation_for_carotenoids_and_dry_matter_contents_in_cassava_roots/links/0deec53c90f0a40a26000000/Sampling-variation-for-carotenoids-and-dry-matter-contents-in-cassava-roots.pdf

[77] Schafer R. What Is a Savitzky-Golay Filter? [Lecture Notes]. IEEE Signal Process Mag. 2011;28(4):111–7. doi: 10.1109/msp.2011.941097

[78] Czarnecki MA. Resolution enhancement in second-derivative spectra. Appl Spectrosc. 2015;69(1):67–74. doi: 10.1366/14-07568 25499557

[79] Jimenez R, Taslakov M, Jeanneret F, Hofstetter D, Beck M, Faist J, et al. Ozone detection by differential absorption spectroscopy at ambient pressure with a 9.6?m pulsed quantum-cascade laser. Applied Physics B: Lasers and Optics. 2004;78(2):249–56. doi: 10.1007/s00340-003-1358-5

[80] Luo J, Ying K, He P, Bai J. Properties of Savitzky–Golay digital differentiators. Digital Signal Processing. 2005;15(2):122–36. doi: 10.1016/j.dsp.2004.09.008

[81] Barnes RJ, Dhanoa MS, Lister SJ. Standard Normal Variate Transformation and De-Trending of Near-Infrared Diffuse Reflectance Spectra. Appl Spectrosc. 1989;43(5):772–7. doi: 10.1366/0003702894202201

[82] Gholizadeh A, Borůvka L, Saberioon MM, Kozák J, Vašát R, Němeček K. Comparing different data preprocessing methods for monitoring soil heavy metals based on soil spectral features. Soil Water Res. 2015;10(4):218–27. doi: 10.17221/113/2015-swr

[83] Osborne BG, Fearn T, Hindle PH. Practical NIR spectroscopy with applications in food and beverage analysis. 2nd ed. Essex, England: Addison-Wesley Longman Ltd; 1993.

[84] Rinnan Åsmund, Berg F van den, Engelsen SB. Review of the most common pre-processing techniques for near-infrared spectra. TrAC Trends in Analytical Chemistry. 2009;28(10):1201–22. doi: 10.1016/j.trac.2009.07.007

[85] Workman J Jr., Weyer L. Practical Guide and Spectral Atlas for Interpretive Near-Infrared Spectroscopy. CRC Press; 2012. doi: 10.1201/b11894

[86] Honorato FA, Barros Neto B de, Martins MN, Galvão RKH, Pimentel MF. Transferência de calibração em métodos multivariados. Quím Nova. 2007;30(5):1301–12. doi: 10.1590/s0100-40422007000500044

[87] Medeiros DT de, Melo RR de, Cademartori PHG de, Batista FG, Mascarenhas ARP, Scatolino MV, et al. Prediction of the basic density of tropical woods by near-infrared spectroscopy. CERNE. 2023;29. doi: 10.1590/01047760202329013262

[88] Burns DA, Ciurczak EW. Handbook of Near-Infrared Analysis. CRC Press; 2007. doi: 10.1201/9781420007374

[89] Sampaio PS, Castanho A, Almeida AS, Oliveira J, Brites C. Identification of rice flour types with near-infrared spectroscopy associated with PLS-DA and SVM methods. Eur Food Res Technol. 2019;246(3):527–37. doi: 10.1007/s00217-019-03419-5

[90] Sohn S-I, Pandian S, Oh Y-J, Zaukuu J-L, Na C-S, Lee Y-H, et al. Vis-NIR Spectroscopy and Machine Learning Methods for the Discrimination of Transgenic *Brassica napus* L. and Their Hybrids with *B. juncea*. Processes. 2022;10(2):240. doi: 10.3390/pr10020240

[91] Posom J, Maraphum K. Achieving prediction of starch in cassava (*Manihot esculenta* Crantz) by data fusion of Vis-NIR and Mid-NIR spectroscopy via machine learning. Journal of Food Composition and Analysis. 2023;122:105415. doi: 10.1016/j.jfca.2023.105415

[92] Freitas EL, Brito AC, de Oliveira SAS, de Oliveira EJ. Early diagnosis of cassava frog skin disease in powdered tissue samples using near-infrared spectroscopy. Eur J Plant Pathol. 2019;156(2):547–58. doi: 10.1007/s10658-019-01904-x

[93] Wold S, Sjöström M, Eriksson L. PLS-regression: a basic tool of chemometrics. Chemometrics and Intelligent Laboratory Systems. 2001;58(2):109–30. doi: 10.1016/s0169-7439(01)00155-1

[94] Viera AJ, Garrett JM. Understanding interobserver agreement: the kappa statistic. Fam Med. 2005;37(5):360–3. 15883903

[95] Chang C-W, Laird DA, Mausbach MJ, Hurburgh CR. Near‐Infrared Reflectance Spectroscopy–Principal Components Regression Analyses of Soil Properties. Soil Science Soc of Amer J. 2001;65(2):480–90. doi: 10.2136/sssaj2001.652480x

[96] Ritthiruangdej P, Ritthiron R, Shinzawa H, Ozaki Y. Non-destructive and rapid analysis of chemical compositions in Thai steamed pork sausages by near-infrared spectroscopy. Food Chem. 2011;129(2):684–92. doi: 10.1016/j.foodchem.2011.04.110 30634287

[97] Morellos A, Pantazi X-E, Moshou D, Alexandridis T, Whetton R, Tziotzios G, et al. Machine learning based prediction of soil total nitrogen, organic carbon and moisture content by using VIS-NIR spectroscopy. Biosystems Engineering. 2016;152:104–16. doi: 10.1016/j.biosystemseng.2016.04.018

[98] Zhao Y, Zhu Y, Ren Y, Lu Y, Yu C, Chen G, et al. Predictive Modeling of Lignocellulosic Content in Crop Straws Using NIR Spectroscopy. Plants. 2025;14(10):1430. doi: 10.3390/plants14101430 40430995 PMC12114956

[99] Nakawajana N, Posom J. Comparison of Analytical Ability of PLS and SVM Algorithm in Estimation of Moisture Content, Higher Heating Value, and Lower Heating Value of Cassava Rhizome Ground using FT-NIR Spectroscopy. IOP Conf Ser: Earth Environ Sci. 2019;301(1):012032. doi: 10.1088/1755-1315/301/1/012032

[100] Gürbüz B, Aras E, Güz AM, Kahriman F. Prediction performance of NIR calibration models developed with different chemometric techniques to predict oil content in a single kernel of maize. Vibrational Spectroscopy. 2023;126:103528. doi: 10.1016/j.vibspec.2023.103528

[101] Font R, del Río-Celestino M, de Haro-Bailón A. The use of near-infrared spectroscopy (NIRS) in the study of seed quality components in plant breeding programs. Industrial Crops and Products. 2006;24(3):307–13. doi: 10.1016/j.indcrop.2006.06.012

[102] Osborne BG. Applications of near Infrared Spectroscopy in Quality Screening of Early-Generation Material in Cereal Breeding Programmes. Journal of Near Infrared Spectroscopy. 2006;14(2):93–101. doi: 10.1255/jnirs.595

[103] Lane HM, Murray SC, Montesinos-López OA, Montesinos-López A, Crossa J, Rooney DK, et al. Phenomic selection and prediction of maize grain yield from near-infrared reflectance spectroscopy of kernels. The Plant Phenome J. 2020;3: e20002. doi: 10.1002/ppj2.20002

[104] Landis JR, Koch GG. The Measurement of Observer Agreement for Categorical Data. Biometrics. 1977;33(1):159. doi: 10.2307/2529310 843571

[105] Ehounou AE, Cornet D, Desfontaines L, Marie-Magdeleine C, Maledon E, Nudol E, et al. Predicting quality, texture and chemical content of yam (*Dioscorea alata* L.) tubers using near infrared spectroscopy. Journal of Near Infrared Spectroscopy. 2021;29(3):128–39. doi: 10.1177/09670335211007575

[106] Peternelli LA, Gonçalves MTV, Fernandes JG, Brasileiro BP, Teófilo RF. Selection of sugarcane clones via multivariate models using near-infrared (NIR) spectroscopy data. Aust J Crop Sci. 2020;(14(6):2020):889–96. doi: 10.21475/ajcs.20.14.06.p2099

[107] Abincha W, Ikeogu UN, Kawuki R, Egesi C, Rabbi I, Parkes E, et al. Portable Spectroscopy Calibration with Inexpensive and Simple Sampling Reference Alternatives for Dry Matter and Total Carotenoid Contents in Cassava Roots. Applied Sciences. 2021;11(4):1714. doi: 10.3390/app11041714

[108] Rosales A, Crossa J, Cuevas J, Cabrera-Soto L, Dhliwayo T, Ndhlela T, et al. Near-Infrared Spectroscopy to Predict Provitamin A Carotenoids Content in Maize. Agronomy. 2022;12(5):1027. doi: 10.3390/agronomy12051027

[109] Khongkaew P, Cruz J, Bertotto JP, Cárdenas V, Alcalà M, Nuchtavorn N, et al. A Comparative Study of Benchtop and Portable NIR and Raman Spectroscopic Methods for the Quantitative Determination of Curcuminoids in Turmeric Powder. Foods. 2022;11(15):2187. doi: 10.3390/foods11152187 35892772 PMC9331271

